# Variation in healthcare services utilization and continuity of care in long-term care facilities: a cross-sectional study

**DOI:** 10.1186/s12913-025-13321-4

**Published:** 2025-09-29

**Authors:** Johannes Schwabe, Brian W. Pulling, Gillian E. Caughey, Maria Crotty, Helena Williams, Andrew Kellie, David Roder, Krystal-Lee Nixon, Gillian Harvey, Janet K. Sluggett, Monica Cations, Tiffany K. Gill, Jyoti Khadka, Megan Corlis, Marilyn von Thien, Maria C. Inacio

**Affiliations:** 1 Registry of Senior Australians Research Centre, South Australian Health and Medical Research Institute, North Terrace, Box PO 11060, Adelaide, SA 5001 Australia; 2 Registry of Senior Australians Research Centre, Caring Futures Institute, School of Nursing and Health Sciences, Flinders University, Adelaide, South Australia Australia; 3 UniSA Allied Health and Human Performance, University of South Australia, Adelaide, South Australia Australia; 4 Adelaide Medical School, Faculty of Health and Medical Sciences, The University of Adelaide, Adelaide, South Australia Australia; 5 College of Medicine and Public Health, Flinders University, Adelaide, South Australia Australia; 6 Southern Adelaide Local Health Network, SA Health, Adelaide, South Australia Australia; 7 Silver Chain Group, Adelaide, South Australia Australia; 8 East Adelaide Healthcare, Newton, South Australia Australia; 9 SA Health Dental Service, Adelaide, South Australia Australia; 10 College of Nursing and Health Sciences, Flinders University, Bedford Park, South Australia Australia; 11 College of Education, Psychology and Social Work, Flinders University, Bedford Park, South Australia Australia; 12 Adelaide Nursing School, University of Adelaide, Adelaide, South Australia Australia; 13 Australian Nursing and Midwifery Federation (SA Branch), Adelaide, South Australia Australia

**Keywords:** Long-term care, Primary healthcare, Continuity of patient care, General practice, Health services for the aged

## Abstract

**Background:**

Primary and specialist healthcare services are critical to ensuring high-quality care for people living in long-term residential aged care facilities (LTCFs). In Australia, these government-subsidized services include general practitioner attendances, health assessments, management plans, allied health services, pain medicine specialists, and mental healthcare, among others. Although the utilization of these services is known to be suboptimal, the extent and nature of variation in their use across LTCFs nationally remain unknown. Importantly, variation that is not attributable to resident needs or planned system design—termed *unwarranted variation*—has been shown to negatively impact health outcomes and warrants investigation. To address this gap, this population-based study aims to examine the national variation in primary and selected specialist healthcare services utilization and continuity of care in residential aged care facilities and characteristics of facility utilization outliers.

**Methods:**

A national cross-sectional study of 173,275 non-Indigenous residents aged ≥65 years from 2,744 Australian facilities in 2019 was conducted. To evaluate continuity of care, the cohort was restricted to LTCF residents who entered care in 2019 and were alive for at least six months (*N* = 41,654 individuals in 2,680 LTCFs). Adjusted median service and continuity of care utilization per 100 residents were calculated. National variation in the rate of healthcare service utilization was quantified using inlier-ranges, categorized as minimal = 0, low < 20, moderate = 20–79, high = 80–99, and maximal = 100.

**Results:**

Maximal variation for services with moderate utilization (median = 22.1–60.6/100 residents) was observed for after-hours attendances, urgent after-hours attendances, health assessments, management plans, podiatry, and optometric services. Continuity of care had low-to-moderate utilization (median = 13.4–26.6/100) and moderate-to-high variation (inlier-range = 68–95.5). Some services had high (median = 99.9/100, general attendances) or low (median < 8.2/100, specialist attendances) utilization and low-to-moderate variation. A small number of mostly high-utilization outliers were identified.

**Conclusions:**

There is substantial variation in utilization of healthcare services and continuity of care amongst residential aged care facilities nationally. While some facilities deliver high levels of preventive and disease management healthcare services supporting residents to have high continuity of care, many facilities face challenges facilitating access to adequate healthcare for their residents.

**Supplementary Information:**

The online version contains supplementary material available at 10.1186/s12913-025-13321-4.

## Background

In Australia, 1.5 million people accessed Commonwealth-funded aged care services between 2022 and 2023, with 250,273 accessing permanent care in long term care facilities (LTCFs) [1]. Due to their complex healthcare needs, complicated by frailty and polypharmacy, individuals in LTCFs critically depend on high-quality primary healthcare. Between 2016 and 2017, it was estimated that 96–98% of LTCF residents visited a general practitioner (GP) an average of 16–25 times per year [2, 3]. In Australia, most GPs operate from community-based practices rather than being directly employed by LTCFs, and they routinely make scheduled visits to provide services [4]. This model contrasts with approaches that rely more on facility-based physicians, as seen in the United States. Recent work has shown that certain primary healthcare services are associated with better outcomes for residents, specifically lower risks of hospitalizations and mortality [5]. For example, preventive and disease management services, specifically when used together, and high continuity of care with one’s providers, offer the most benefit to residents [5]. However, while it is known that the utilization of some health services may not meet the needs of LTCF residents [6], less is understood about the variation in healthcare service delivery nationally and whether certain LTCF characteristics are associated with service utilization.

Variation in the utilization of healthcare services reflects quality of care and is influenced by healthcare needs of the population of interest, system-level factors [7], and consumer preferences [8]. Explanations for healthcare service variation include capacity or access (i.e., workforce factors), evidence (e.g. uptake of clinical practice guidelines), agency (e.g. patient or clinician preference), among others [9]. While a moderate or even high amount of variation in healthcare service utilization is not necessarily a problem when it can be ascribed to appropriate health system features, it may also be considered *unwarranted* variation, if disagreement regarding best practices, inequitable access, poor uptake of clinical practice guidelines, supply-induced demand, or other unjustified sources are causing it [10]. For example, in Australia, only a third of people entering a LTCF receive a comprehensive health assessment, despite this service being available to all new residents [11]. Therefore, it is plausible that the observed variation in comprehensive health assessment utilization is due to sources representing unwarranted variation.

Unwarranted variation in healthcare service utilization can cause either real harm from not receiving necessary care, or potential harm from receiving unnecessary care. In either case, individuals are exposed to non-evidence-based care, whereby they receive (or do not receive) appropriate care [12]. Efforts to improve the quality of LTCFs thereby need a clear understanding of the population-level variation in utilization of healthcare services in this setting, but this is lacking. In Australia, evidence on variation in healthcare service utilization (particularly GP services) has largely focused on differences between geographical areas in the general population [13–15]. Similarly, in the Australian Atlas of Healthcare Variation, variation in the utilization of medication reviews and GP mental health plans did not specifically evaluate older people in LTCFs [16]. One notable study examined variation in the provision of collaborative medication reviews to new entrants of LTCFs [17]. However, the extent of variation experienced by the growing population receiving aged care services in LTCFs more generally, is unknown. Therefore, an evaluation of the variation in utilization of healthcare services based on characteristics of LTCFs to identify target areas for improvement nationally is warranted.

The aims of this study were to: (1) examine the national variation between LTCFs in healthcare service utilization and continuity of GP care; (2) describe differences in variation by LTCF ownership, location, and size; and (3) identify low or high outliers of service utilization and continuity of GP care and their characteristics.

## Methods

### Study design, setting and data sources

A population-based cross-sectional study was conducted using the Registry of Senior Australians (ROSA) National Historical Cohort [18]. This data source has been previously described [18]. Briefly, at the time of this study ROSA contained integrated national and state-based health, aged care and social welfare information for approximately 3.5 million people who have accessed aged care services between 2002 and 2020. The ROSA datasets used in this study included: aged care eligibility assessments for permanent, long-term home, respite or transition care services; episodes of care service records; entry into permanent care needs assessments; pharmaceutical dispensing records (Pharmaceutical Benefits Scheme; PBS); subsidized healthcare service records (Medicare Benefits Schedule; MBS); and deaths (National Death Index; NDI).

### Study cohort

National permanent LTCF residents aged ≥ 65 years old, who spent at least one day in care in 2019, did not identify as Aboriginal or Torres Strait Islander, and were not Department of Veterans’ Affairs card holders were studied (*N* = 173,275 individuals in 2,744 LTCFs). Department of Veterans’ Affair card holders were not included in the cohort due to their concessional entitlements that may lead to a differential level of health care service access. Aboriginal or Torres Strait Islander individuals were not included because this study did not have the necessary leadership and culturally appropriate processes required for inclusion. For the continuity of care evaluation, the cohort was restricted to LTCF residents who entered care in 2019 and were alive for at least six months (*N* = 41,654 individuals in 2,680 LTCFs).

### Healthcare services

Healthcare services subsidized by the Australian government through the publicly funded universal health care insurance scheme (“Medicare”) utilized between 01/01/2019 and 12/31/2019, and continuity of GP care within six months of LTCF entry were examined.

Specific primary and specialist care services analyzed included (Supplemental Table 1 for coding): GP or medical practitioner (MP) general attendances, after-hours attendances, and urgent after-hours attendances; nurse practitioner attendances; GP/MP health assessments, management plans, and attendances associated with practice incentive programs. Allied health services examined included: optometric services, comprehensive medication reviews, and allied health services as part of management plans. Podiatry services represented 94% of the allied health services delivered as part of a management plan and were therefore evaluated separately. Selected specialist attendances of clinical importance to the older population were also included: pain medicine, geriatric medicine, and multimorbidity attendances. Mental healthcare services included: psychiatry attendances, psychological therapy, focused psychological strategies, and GP mental health attendances.

Continuity of GP care was based on utilization of health services delivered by a GP or MP (but not specialists) and determined by comparing the most frequently seen practitioner within the first six months of LTCF entry to those seen in the 24 months preceding entry (Supplemental Table 1). Continuity of care was classified as (i) new GP/MP (i.e., most frequently seen after entry was never seen prior to entry), (ii) known GP/MP (i.e., most frequently seen after entry was seen at least once prior to entry), and (iii) usual GP/MP (i.e., most frequently seen did not change from before to after entry) as previously defined [19]. Continuity of care during the transition into residential care is critical for patient outcomes; therefore, an established ‘before-after’ measure specifically developed in the context of LTCFs to capture whether the primary care relationship was maintained during this pivotal period was employed [19].

### LTCF characteristics

The following LTCF characteristics were ascertained from the LTCF episodes of care and included: size (i.e., number of residents during the year studied), ownership type (i.e., government, not-for-profit, private), remoteness (i.e., metropolitan, non-metropolitan). Location is reported as a descriptive characteristic (by Australian state and territory: Australian Capital Territory, New South Wales, Northern Territory, Queensland, South Australia, Tasmania, Victoria, Western Australia).

### Covariates

Age (categories 65–69, 70–74, 75–79, 80–84, 85–89, ≥90 years old), sex, RxRisk-V [20] comorbidity score (0–2, 3–5, 6–10, ≥11) with a 6-month look-back period at LTCF entry, dementia diagnosis (ascertained from medications, or aged care eligibility or care needs assessments) [21, 22], and mobility care needs (0–3, 3 = most dependent) were examined. While the latter two are clinically important and were used for cohort description, they were not included in the risk adjustment, as in line with the Dartmouth Atlas of Health Care and the Australian Atlas of Healthcare Variation approaches [6, 13].

### Analysis

Descriptive statistics characterized the individuals and LTCFs examined. Crude and adjusted median healthcare utilization and continuity of care rates per 100 residents were calculated. To obtain risk-adjusted rates, individual predicted probabilities for receiving the service of interest (or continuity of care category) were obtained from logistic regression models adjusted for age, sex, and number of health conditions. These predicted probabilities were summed across all residents to yield the total expected number of events in the LTCF. Dividing the observed count by the expected count yields an observed-to-expected ratio, which, when multiplied by the overall crude rate and then by 100, produces the risk-adjusted rate per 100 residents. To facilitate interpretation, the adjusted utilization of a service per 100 residents was capped at 100. After risk adjustment, capping is achieved by restricting any values above 100 to 100. This step corrects an artefact that can occur when LTCFs with maximal utilization are adjusted. The utilization rates range between 0 and 100 and can be interpreted as the prevalence per 100 residents. Given a small number of missing cases (11 of 2,744 LTCFs, 0.4%), a complete case analysis was conducted. Other than having missing values for the remoteness classification, the excluded LTCFs did not differ notably in case mix or service utilization from the included LTCFs. Variation in the rate of service utilization was examined by looking at the *inlier-range* from the boxplot methodology [23]. To derive *inlier-range*, the lower and upper bounds of inliers are calculated as Quartile 1-1.5*interquartile-range (IQR) and Quartile 3 + 1.5*interquartile-range, respectively. The *inlier-range* also defines inlier values in this study. LTCFs with utilization outside of the *inlier-range* are defined as outliers. As a measure of variation, the *inlier-range* was evaluated categorically (minimal = 0, low = 1–19, moderate = 20–79, high = 80–99, and maximal = 100). The categories were derived from exploratory data analysis and clinical judgment. We additionally report *IQR* and median absolute deviations in tabular form. To visualize the variation and identify LTCF outliers, we combined box-whisker-plots and strip charts. Where LTCF outliers were identified, they are reported by total number and as a proportion of all LTCFs. Outliers by LTCF size, ownership type, and remoteness are reported in total number and as a proportion of all service-specific outliers. All analyses were conducted using R v4.3.0 (R Core Team, Vienna, Austria).

## Results

### Cohort

The cohort (*N* = 173,275) had a median age of 84 years (IQR = 79–89), 64.0% (*n* = 110,929) were female, 81.3% (*n* = 141,050) had a comorbidity score ≥3, and 50.6% (*n* = 87,648) were living with dementia (Table 1). Of 2,744 LTCFs examined, 57.2% (*n* = 1,570) were not-for-profit, 62.8% (*n* = 1,723) were in metropolitan areas, and 53.7% (*n* = 1,471) had fewer than 60 residents (Table 1). The characteristics of the cohort in which continuity of care was examined were similar (Table 1).

**Table 1:** Study cohort and Long-Term care facility characteristics

Variable	Categories	Overall Cohort *N* (%)	Continuity of Care Cohort *N* (%)
Resident Characteristics	N	173,275	41,768
Sex (%)	Female	110,929 (64.0)	25,592 (61.3)
	Male	62,346 (36.0)	16,176 (38.7)
Median age at entry [IQR]		84 [79, 89]	85 [79, 89]
Number of health conditions (%)	0–2	32,225 (18.6)	7758 (18.6)
	3–5	70,420 (40.6)	17,062 (40.8)
	6–10	66,735 (38.5)	16,003 (38.3)
	11+	3895 (2.25)	945 (2.26)
Mobility Level of Need (%)	0 - None	4644 (2.68)	763 (1.83)
	1 - Low	7075 (4.08)	904 (2.16)
	2 - Medium	77,439 (44.8)	18,217 (43.6)
	3 - High	83,703 (48.4)	21,880 (52.4)
Dementia (%)		87,648 (50.6)	21,511 (51.5)
State (%)	Australian Capital Territory	2184 (1.26)	529 (1.27)
	New South Wales	56,670 (32.7)	13,453 (32.2)
	Northern Territory	311 (0.18)	72 (0.17)
	Queensland	32,230 (18.6)	8094 (19.4)
	South Australia	16,124 (9.31)	3851 (9.22)
	Tasmania	4398 (2.53)	1046 (2.50)
	Victoria	46,650 (26.9)	11,003 (26.3)
	Western Australia	14,708 (8.49)	3720 (8.91)
Remoteness (%)	Metropolitan	121,122 (69.9)	29,249 (70.0)
	Non-Metropolitan	51,664 (29.8)	12,405 (29.7)
	missing	489 (0.28)	114 (0.27)
Ownership type (%)	Government	6532 (3.77)	1570 (3.76)
	Not-For-Profit	95,758 (55.3)	22,708 (54.4)
	Private	70,985 (41.0)	17,490 (41.9)
Facility size (%)	1–29	9402 (5.43)	2189 (5.24)
	30–59	42,039 (24.3)	10,083 (24.1)
	60–89	46,982 (27.1)	11,668 (27.9)
	90+	74,852 (43.2)	17,828 (42.7)
LTCF Characteristics	N	2744	2690
Facility size (%)	1–29	520 (19.0)	474 (17.6)
	30–59	951 (34.7)	946 (35.2)
	60–89	633 (23.1)	631 (23.5)
	90+	640 (23.3)	639 (23.8)
State (%)	Australian Capital Territory	25 (0.91)	24 (0.89)
	New South Wales	889 (32.4)	878 (32.6)
	Northern Territory	10 (0.36)	8 (0.30)
	Queensland	475 (17.3)	465 (17.3)
	South Australia	249 (9.11)	243 (9.03)
	Tasmania	72 (2.62)	72 (2.68)
	Victoria	776 (28.3)	762 (28.3)
	Western Australia	248 (9.03)	238 (8.85)
Remoteness (%)	Metropolitan	1723 (62.8)	1691 (62.9)
	Non-Metropolitan	1010 (37.2)	989 (36.8)
	missing	11 (0.40)	10 (0.37)
Ownership type (%)	Government	236 (8.67)	230 (8.55)
	Not-For-Profit	1570 (57.2)	1537 (57.1)
	Private	938 (34.2)	923 (34.3)

### Variation in healthcare service utilization and continuity of care

**Fig. 1:**
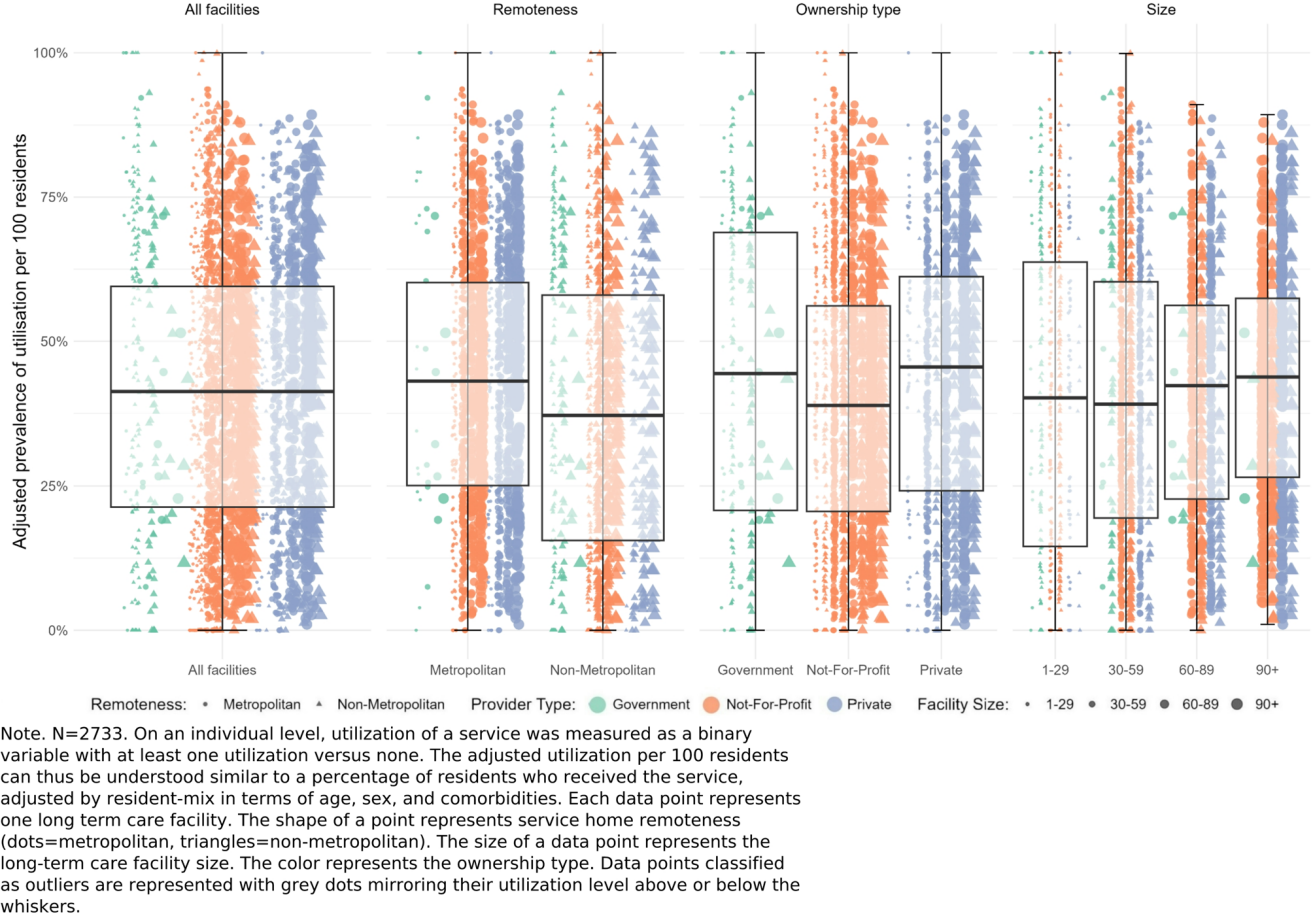
Adjusted utilization of health assessments per 100 residents of long-term care facilities by facility characteristics. Note. *N* = 2733. On an individual level, utilization of a service was measured as a binary variable with at least one utilization versus none. The adjusted utilization per 100 residents can thus be understood similar to a percentage of residents who received the service, adjusted by resident-mix in terms of age, sex, and comorbidities. Each data point represents one long term care facility. The shape of a point represents service home remoteness (dots = metropolitan, triangles = non-metropolitan). The size of a data point represents the long-term care facility size. The color represents the ownership type. Data points classified as outliers are represented with grey dots mirroring their utilization level above or below the whiskers

**Fig. 2:**
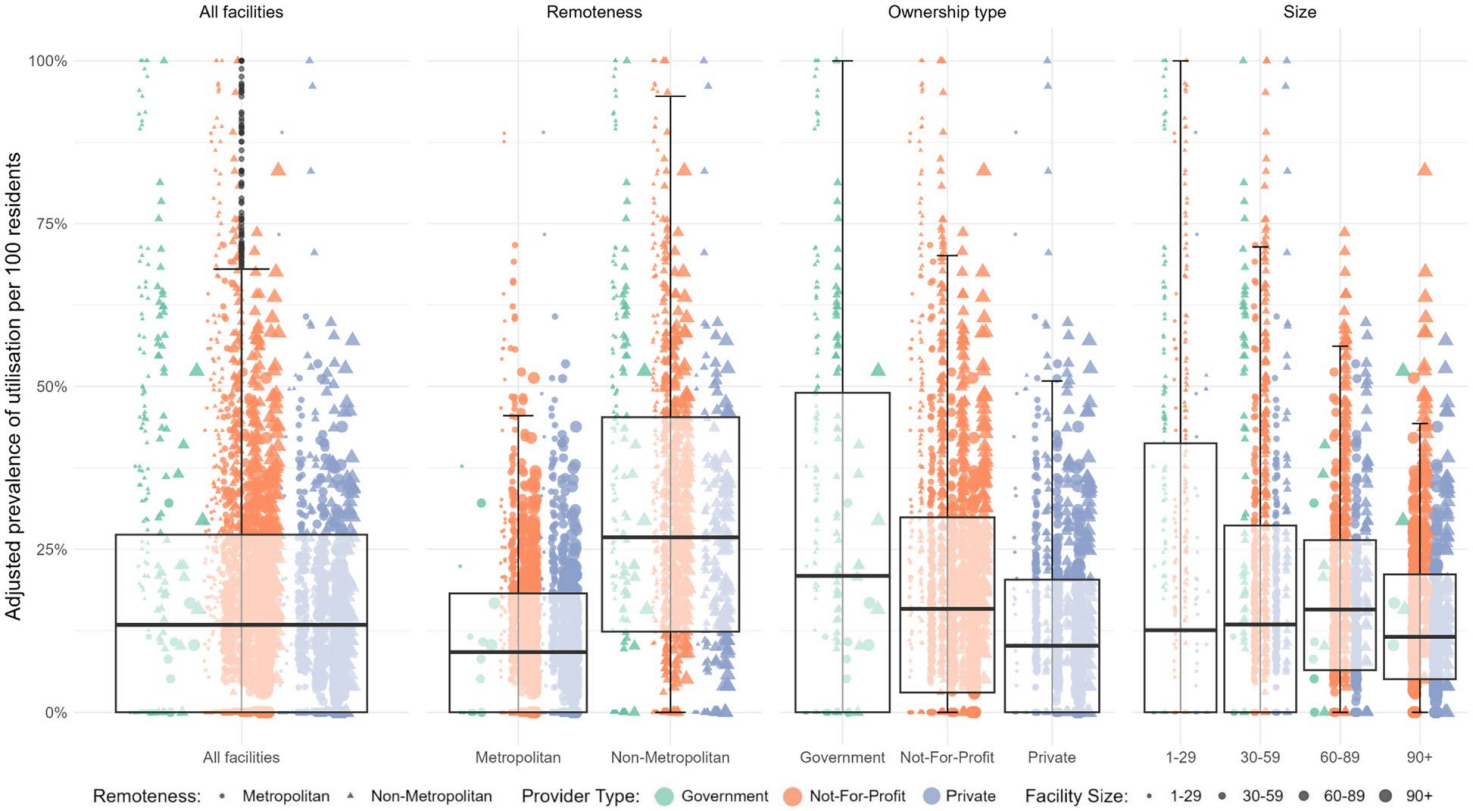
Adjusted prevalence of continuity of care per 100 residents by long-term care facility characteristics. Note. *N* = 2680. On an individual level, prevalence of continuity of care– usual GP was measured as a binary variable such that care continued after entry into long term care with the most frequently seen GP before entry, or not. The adjusted utilization per 100 residents can thus be understood similar to a percentage of residents who experienced continuity of care, adjusted by resident-mix in terms of age, sex, and comorbidities. Each data point represents one long-term care facility. The shape of a point represents long term care facility remoteness (dots = metropolitan, triangles = non-metropolitan). The size of a data point represents the long-term care facility size. The color represents the ownership type. Data points classified as outliers are represented with grey dots mirroring their utilization level above or below the whiskers

**Fig. 3:**
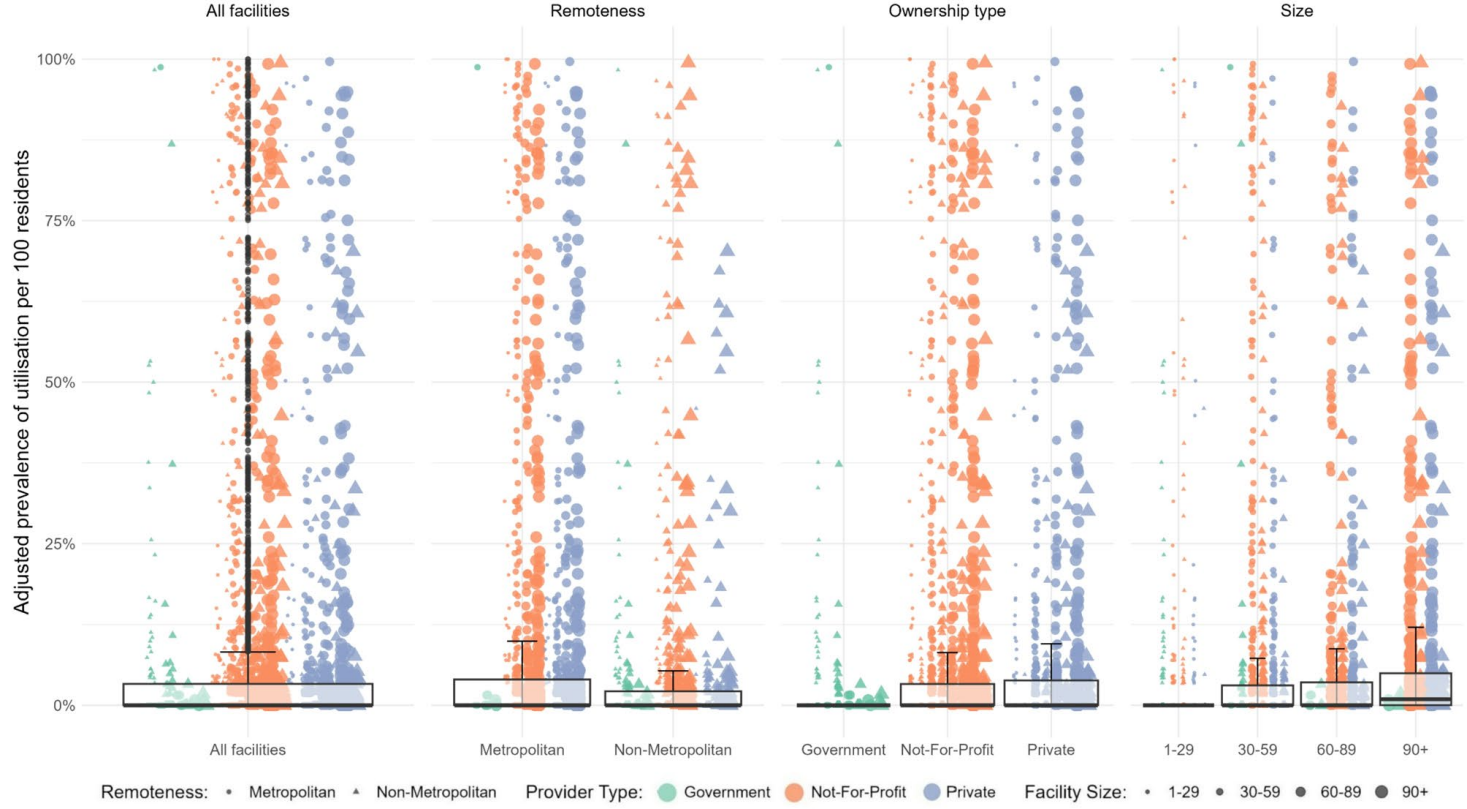
Adjusted utilization of nurse practitioners per 100 residents of long-term care facilities by facility characteristics. Note. *N*=2733. On an individual level, utilization of a service was measured as a binary variable with at least one utilization versus none. The adjusted utilization per 100 residents can thus be understood similar to a percentage of residents who received the service, adjusted by resident-mix in terms of age, sex, and comorbidities. Each data point represents one long term care facility. The shape of a point represents long term care facility remoteness (dots=metropolitan, triangles=non-metropolitan). The size of a data point represents the long-term care facility size. The color represents the ownership type. Data points classified as outliers are represented with grey dots mirroring their utilization level above or below the whiskers

All estimates of healthcare service utilization and variation reported below are risk-adjusted for age, sex, and the number of health conditions. After-hours GP/MP attendances had a high utilization (60.6/100 residents), maximal variation (*inlier-range* = 100 [0;100]), with no outliers identified (Table 2, Supplemental Fig. 1). Urgent after-hours GP/MP attendances had a moderate utilization (22.1/100 residents), maximal variation (*inlier-range* = 100 [0;100]), and no outliers were identified (Table 2, Supplemental Fig. 2). GP/MP health assessments had a moderate utilization (41.3/100 residents), maximal variation (*inlier-range* = 100 [0;100]), and no outliers identified (Table 2; Fig. 1). GP/MP management plans had a moderate utilization (56.7/100 residents), maximal variation (*inlier-range* = 100 [0;100]), and no outliers were identified (Table 2, Supplemental Fig. 3). Podiatry services had a moderate utilization (30.2/100 residents), maximal variation (*inlier-range =* 100 [0;100]), and no outliers were identified (Table 2, Supplemental Fig. 4). Optometric services had a moderate utilization (39.6/100 residents), maximal variation (*inlier-range* = 100 [0;100]), and no outliers were identified (Table 2, Supplemental Fig. 5). Comprehensive medication reviews had a moderate utilization (33.1/100 residents), high variation (*inlier-range* = 84.8 [0;84.8]), and 0.44% (12/2733) LTCFs outside of the *inlier-range* (Table 2, Supplemental Fig. 6). Geriatric specialists had a low utilization, moderate variation, and 4.81% (132/2733) of LTCFs were outliers (Table 2, Supplemental Fig. 7). Multimorbidity specialists had a low utilization, moderate variation, and 3.57% (98/2733) of LTCFs were outliers (Table 2, Supplemental Fig. 8). Prevalence of continuity of care with the usual GP/MP was low, varied moderately, and 2.95% (81/2680) of LTCFs were outliers (Table 2; Fig. 2). Similarly, prevalence of continuity of care with a known GP/MP was low (9.9/100 residents), varied moderately, and 2.13% (84/2680) of LTCFs were outliers (Table 2, Supplemental Fig. 9). General GP/MP attendances had a very high utilization, low variation, and 8.64% (237/2733) of LTCFs were outliers (Table 2, Supplemental Fig. 10). Nurse practitioner attendances, GP/MP attendances associated with the Practice Incentives Program (PIP), pain specialists, and mental health services all had low utilization and low variation with few outliers (Table 2; Fig. 3, Supplemental Figs. 11 to 16).

**Table 2:** Metrics of central tendency and dispersion for healthcare service utilization of long-term care facilities and outlier characteristics

Metric	All facilities	General attendances			After hours			Urgent after hours			Nurse practitioners		
Total number of facilities	2744	2733			2733			2733			2733		
Median [Q1; Q3]		99.9 [98.3; 100]			60.6 [33.7; 82.7]			22.1 [5.89; 50.4]			0 [0; 3.30]		
IQR		1.71			48.9			44.5			3.30		
Median Absolute Deviation		0.04			24.5			19.3			0		
Inlier-range [b_lower_; b_upper_]^2^		4.25 [95.7; 100]			100 [0; 100]			100 [0; 100]			8.24 [0; 8.24]		
Outliers total (% of all LTCF)		237 (8.64)			-			-			463 (16.9)		
Facility characteristics	N (%)	Outliers, N (% of total outliers)	Median	Inlier-range [b_lower_; b_upper_]^2^	Outliers, N (% of total outliers)	Median	Inlier-range [b_lower_; b_upper_]^2^	Outliers, N (% of total outliers)	Median	Inlier-range [b_lower_; b_upper_]^2^	Outliers, N (% of total outliers)	Median	Inlier-range [b_lower_; b_upper_]^2^
Ownership type													
Government	236 (8.64)	27 (11.4)	100	0 [100; 100]	-	49.7	100 [0; 100]	-	12.9	70 [0; 70]	23 (4.97)	0	0 [0; 0]
Not-For-Profit	1570 (57.2)	134 (56.5)	100	4 [96; 100]	-	53.3	100 [0; 100]	-	16.7	100 [0; 100]	276 (59.6)	0	8 [0; 8]
Private	938 (34.2)	76 (32.1)	99.9	4 [96; 100]	-	75.5	100 [0; 100]	-	38.3	100 [0; 100]	164 (35.4)	0	10 [0; 10]
Remoteness					-			-					
Metropolitan	1723 (62.8)	132 (55.7)	100	4 [96; 100]	-	74.6	100 [0; 100]	-	40.0	100 [0; 100]	333 (71.9)	0	10 [0; 10]
Non-Metropolitan	1021 (37.2)	105 (44.3)	100	4 [96; 100]	-	34.9	100 [0; 100]	-	6.0	36 [0; 36]	130 (28.1)	0	5 [0; 5]
Size					-			-					
1–29	520 (19.0)	60 (25.3)	100	0 [100; 100]	-	48.1	100 [0; 100]	-	11.1	99 [0; 99]	66 (14.3)	0	0 [0; 0]
30–59	951 (34.7)	82 (34.6)	100	5 [95; 100]	-	57.7	100 [0; 100]	-	22.2	100 [0; 100]	146 (31.5)	0	8 [0; 8]
60–89	633 (23.1)	48 (20.3)	99.9	4 [96; 100]	-	63.1	100 [0; 100]	-	23.2	100 [0; 100]	115 (24.8)	0	9 [0; 9]
90 +	640 (23.3)	47 (19.8)	99.1	5 [95; 100]	-	70.6	100 [0; 100]	-	30.8	100 [0; 100]	136 (29.4)	0.9	12 [0; 12]
State													
New South Wales	889 (32.4)	88 (37.1)	100	5 [95; 100]	-	52.9	100 [0; 100]	-	14.0	92 [0; 92]	85 (18.4)	0	3 [0; 3]
Other^4^	107 (3.92)	10 (4.22)	99.3	5 [95; 100]	-	49.0	100 [0; 100]	-	6.0	29 [0; 29]	33 (7.13)	1.3	30 [0; 30]
Queensland	475 (17.3)	38 (16.0)	99.9	4 [96; 100]	-	60.3	100 [0; 100]	-	21.7	100 [0; 100]	104 (22.5)	0	11 [0; 11]
South Australia	249 (9.11)	15 (6.33)	100	3 [97; 100]	-	77.0	100 [0; 100]	-	40.1	100 [0; 100]	36 (7.77)	0	6 [0; 6]
Victoria	776 (28.3)	70 (29.5)	100	4 [96; 100]	-	73.0	100 [0; 100]	-	44.3	100 [0; 100]	102 (22)	0	8 [0; 8]
Western Australia	248 (9.07)	16 (6.75)	100	4 [96; 100]	-	47.6	100 [0; 100]	-	13.6	60 [0; 60]	103 (22.2)	4.0	100 [0; 100]

Metric	All facilities	Health assessments			Management plans			Attendances associated with PIP					
Total number of facilities	2744	2733			2733			2733					
Median [Q1; Q3]		41.3 [21.3; 59.5]			56.7 [34.3; 75.0]			0 [0; 1.77]					
IQR		38.2			40.7			1.77					
Median Absolute Deviation		19.1			20.2			0					
Inlier-range [b_lower_; b_upper_]^2^		100 [0; 100]			100 [0; 100]			4.38 [0; 4.38]					
Outliers total (% of all LTCF)		-			-			217 (7.91)					
Facility characteristics	N (%)	Outliers, N (% of total outliers)	Median	Inlier-range [b_lower_; b_upper_]^2^	Outliers, N (% of total outliers)	Median	Inlier-range [b_lower_; b_upper_]^2^	Outliers, N (% of total outliers)		Median		Inlier-range [b_lower_; b_upper_]^2^	
Ownership type													
Government	236 (8.64)	-	44.4	100 [0; 100]	-	42.9	100 [0; 100]	24 (11.1)		0		0 [0; 0]	
Not-For-Profit	1570 (57.2)	-	38.9	100 [0; 100]	-	54.6	100 [0; 100]	126 (58.1)		0		5 [0; 5]	
Private	938 (34.2)	-	45.6	100 [0; 100]	-	63.3	100 [0; 100]	67 (30.9)		0		4 [0; 4]	
Remoteness													
Metropolitan	1723 (62.8)	-	43.1	100 [0; 100]	-	62.9	100 [0; 100]	117 (53.9)		0		4 [0; 4]	
Non-Metropolitan	1021 (37.2)	-	37.2	100 [0; 100]	-	43.5	100 [0; 100]	100 (46.1)		0		5 [0; 5]	
Size													
1–29	520 (19.0)	-	40.2	100 [0; 100]	-	52.3	100 [0; 100]	63 (29.0)		0		0 [0; 0]	
30–59	951 (34.7)	-	39.1	100 [0; 100]	-	56.1	100 [0; 100]	75 (34.6)		0		5 [0; 5]	
60–89	633 (23.1)	-	42.3	100 [0; 100]	-	56.7	100 [0; 100]	39 (18.0)		0		4 [0; 4]	
90 +	640 (23.3)	-	43.8	100 [0; 100]	-	59.3	100 [0; 100]	40 (18.4)		0		4 [0; 4]	
State													
New South Wales	889 (32.4)	-	33.1	100 [0; 100]	-	57.5	100 [0; 100]	56 (25.8)		0		4 [0; 4]	
Other^4^	107 (3.92)	-	29.0	92 [0; 92]	-	40.7	100 [0; 100]	8 (3.69)		0		5 [0; 5]	
Queensland	475 (17.3)	-	49.4	100 [0; 100]	-	55.9	100 [0; 100]	40 (18.4)		0		4 [0; 4]	
South Australia	249 (9.11)	-	39.9	100 [0; 100]	-	58.5	100 [0; 100]	14 (6.45)		0		4 [0; 4]	
Victoria	776 (28.3)	-	47.1	100 [0; 100]	-	59.1	100 [0; 100]	89 (41.0)		0		5 [0; 5]	
Western Australia	248 (9.07)	-	42.9	100 [0; 100]	-	50.6	100 [0; 100]	10 (4.61)		0		3 [0; 3]	

Metric	All facilities	Podiatry			Optometric Services			Comprehensive medication reviews					
Total number of facilities	2733	2733			2733			2733					
Median [Q1; Q3]		30.2 [11; 68.8]			39.6 [22.1; 61.6]			33.1 [19.9; 45.8]					
Inter-Quartile-Range		57.8			39.5			27.0					
Median Absolute Deviation		23.5			19.3			13.3					
Inlier-range [b_lower_; b_upper_]^2^		100 [0; 100]			100 [0; 100]			84.8 [0; 84.8]					
Outliers total (% of all LTCF)		-			-			12 (0.44)					
Facility Characteristics	N (%)	Outliers, N (% of total outliers)	Median	Inlier-range [b_lower_; b_upper_]^2^	Outliers, N (% of total outliers)	Median	Inlier-range [b_lower_; b_upper_]^2^	Outliers, N (% of total outliers)		Median		Inlier-range [b_lower_; b_upper_]^2^	
Ownership type													
Government	236 (8.64)	-	17.7	100 [0; 100]	-	30.6	100 [0; 100]	^1^		26.9		98 [0; 98]	
Not-For-Profit	1570 (57.2)	-	29.3	100 [0; 100]	-	33.6	100 [0; 100]	^1^		33.1		87 [0; 87]	
Private	938 (34.2)	-	36.5	100 [0; 100]	-	53.2	100 [0; 100]	^1^		34.1		83 [0; 83]	
Remoteness													
Metropolitan	1723 (62.8)	-	41.8	100 [0; 100]	-	51.0	100 [0; 100]	^1^		34.7		83 [0; 83]	
Non-Metropolitan	1021 (37.2)	-	19.1	100 [0; 100]	-	25.2	74 [0; 74]	^1^		29.6		90 [0; 90]	
Size													
1–29	520 (19.0)	-	28.0	100 [0; 100]	-	31.9	100 [0; 100]	^1^		27.4		100 [0; 100]	
30–59	951 (34.7)	-	37.8	100 [0; 100]	-	40.7	100 [0; 100]	^1^		34.1		93 [0; 93]	
60–89	633 (23.1)	-	26.4	100 [0; 100]	-	39.0	100 [0; 100]	^1^		33.9		80 [0; 80]	
90 +	640 (23.3)	-	27.8	100 [0; 100]	-	46.3	100 [0; 100]	^1^		33.3		74 [0; 74]	
State													
New South Wales	889 (32.4)	-	41.8	100 [0; 100]	-	37.2	100 [0; 100]	^1^		33.5		82 [0; 82]	
Other^4^	107 (3.92)	-	10.3	41 [0; 41]	-	20.9	48 [0; 48]	^1^		38.9		100 [0; 100]	
Queensland	475 (17.3)	-	14.1	100 [0; 100]	-	44.4	100 [0; 100]	^1^		34.7		85 [0; 85]	
South Australia	249 (9.11)	-	63.1	100 [0; 100]	-	24.8	58 [0; 58]	^1^		30.7		80 [0; 80]	
Victoria	776 (28.3)	-	36.2	100 [0; 100]	-	57.2	100 [0; 100]	^1^		35.8		91 [0; 91]	
Western Australia	248 (9.07)	-	14.3	100 [0; 100]	-	31.6	88 [0; 88]	^1^		18.5		74 [0; 74]	

Metric	All facilities	Specialists – Pain			Specialists – Geriatric			Specialists- Multimorbidity					
Total number of facilities	2733	2733			2733			2733					
Median [Q1; Q3]		0 [0; 1.55]			5.94 [1.33; 16.3]			8.2 [4.23; 13.5]					
Inter-Quartile-Range		1.55			15.0			9.24					
Median Absolute Deviation		0			5.94			4.46					
Inlier-range [b_lower_; b_upper_]^2^		3.88 [0; 3.88]			38.7 [0; 38.7]			27.2 [0; 27.2]					
Outliers total (% of all LTCF)		208 (7.58)			132 (4.81)			98 (3.57)					
Facility characteristics	N (%)	Outliers, N (% of total outliers)	Median	Inlier-range [b_lower_; b_upper_]^2^	Outliers, N (% of total outliers)	Median	Inlier-range [b_lower_; b_upper_]^2^	Outliers, N (% of total outliers)		Median		Inlier-range [b_lower_; b_upper_]^2^	
Ownership type													
Government	236 (8.64)	^1^	0	0 [0; 0]	12 (9.09)	1.5	20 [0; 20]	^1^		3.6		21 [0; 21]	
Not-For-Profit	1570 (57.2)	121 (58.2)	0	4 [0; 4]	46 (34.8)	4.9	30 [0; 30]	^1^		8.1		26 [0; 26]	
Private	938 (34.2)	^1^	0	5 [0; 5]	74 (56.1)	10.2	55 [0; 55]	59 (60.2)		9.2		29 [0; 29]	
Remoteness			0										
Metropolitan	1723 (62.8)	175 (84.1)	0	5 [0; 5]	117 (88.6)	9.6	49 [0; 49]	81 (82.7)		10.0		29 [0; 29]	
Non-Metropolitan	1021 (37.2)	33 (15.9)	0	0 [0; 0]	15 (11.4)	2.4	18 [0; 18]	17 (17.3)		5.4		21 [0; 21]	
Size													
1–29	520 (19.0)	39 (18.8)	0	0 [0; 0]	26 (19.7)	0	26 [0; 26]	31 (31.6)		4.6		32 [0; 32]	
30–59	951 (34.7)	81 (38.9)	0	5 [0; 5]	43 (32.6)	6.0	38 [0; 38]	28 (28.6)		7.5		26 [0; 26]	
60–89	633 (23.1)	47 (22.6)	0	4 [0; 4]	30 (22.7)	6.3	38 [0; 38]	14 (14.3)		8.8		26 [0; 26]	
90 +	640 (23.3)	41 (19.7)	0.8	4 [0; 4]	33 (25.0)	8.1	44 [0; 44]	25 (25.5)		10.0		25 [0; 25]	
State													
New South Wales	889 (32.4)	78 (37.5)	0	4 [0; 4]	56 (42.4)	10.0	45 [0; 45]	36 (36.7)		10.2		30 [0; 30]	
Other^4^	107 (3.92)	^1^	0	3 [0; 3]	^1^	3.8	42 [0; 42]	^1^		4.5		26 [0; 26]	
Queensland	475 (17.3)	25 (12.0)	0	4 [0; 4]	7 (5.30)	2.3	14 [0; 14]	^1^		6.7		23 [0; 23]	
South Australia	249 (9.11)	^1^	0	0 [0; 0]	^1^	4.9	20 [0; 20]	^1^		6.9		20 [0; 20]	
Victoria	776 (28.3)	61 (29.3)	0	3 [0; 3]	62 (47.0)	11.7	55 [0; 55]	45 (45.9)		8.9		29 [0; 29]	
Western Australia	248 (9.07)	40 (19.2)	1.3	7 [0; 7]	^1^	1.6	10 [0; 10]	^1^		6.1		20 [0; 20]	

Metric	All facilities	Psychiatry			Focused psychological strategies			GP mental health services					
Total number of facilities	2733	2733			2733			2733					
Median [Q1; Q3]		1 [0; 3.01]			0 [0; 0.52]			0 [0; 1.82]					
IQR		3.01			0.52			1.82					
Median Absolute Deviation		0.89			0			0					
Inlier-range [b_lower_; b_upper_]^2^		7.49 [0; 7.49]			1.44 [0; 1.44]			4.52 [0; 4.52]					
Outliers total (% of all LTCF)		254 (9.26)			462 (16.9)			213 (7.76)					
Facility characteristics	N (%)	Outliers, N (% of total outliers)	Median	Inlier-range [b_lower_; b_upper_]^2^	Outliers, N (% of total outliers)	Median	Inlier-range [b_lower_; b_upper_]^2^	Outliers, N (% of total outliers)		Median		Inlier-range [b_lower_; b_upper_]^2^	
Ownership type													
Government	236 (8.64)	10 (3.93)	0	0 [0; 0]	20 (4.33)	0	0 [0; 0]	15 (7.04)		0		0 [0; 0]	
Not-For-Profit	1570 (57.2)	155 (61.0)	0.7	8 [0; 8]	241 (52.2)	0	0 [0; 0]	109 (51.2)		0		4 [0; 4]	
Private	938 (34.2)	89 (35.0)	1.4	8 [0; 8]	201 (43.5)	0	3 [0; 3]	89 (41.8)		0		5 [0; 5]	
Remoteness										0			
Metropolitan	1723 (62.8)	222 (87.4)	1.6	10 [0; 10]	326 (70.6)	0	2 [0; 2]	154 (72.3)		0		5 [0; 5]	
Non-Metropolitan	1010 (37.2)	32 (12.6)	0	4 [0; 4]	136 (29.4)	0	0 [0; 0]	59 (27.7)		0		3 [0; 3]	
Size													
1–29	520 (19.0)	43 (16.9)	0	0 [0; 0]	46 (9.96)	0	0 [0; 0]	65 (30.5)		0		0 [0; 0]	
30–59	951 (34.7)	88 (34.6)	0	7 [0; 7]	175 (37.9)	0	0 [0; 0]	82 (38.5)		0		5 [0; 5]	
60–89	633 (23.1)	69 (27.2)	1.4	8 [0; 8]	137 (29.7)	0	3 [0; 3]	34 (16.0)		1.1		4 [0; 4]	
90 +	640 (23.3)	54 (21.3)	1.2	7 [0; 7]	104 (22.5)	0	2 [0; 2]	32 (15.0)		0.9		5 [0; 5]	
State													
New South Wales	889 (32.4)	133 (52.4)	0.8	10 [0; 10]	150 (32.5)	0	0 [0; 0]	66 (31.0)		0		5 [0; 5]	
Other^4^	107 (3.92)	^1^	0	5 [0; 5]	^1^	0	0 [0; 0]	^1^		0		2 [0; 2]	
Queensland	475 (17.3)	48 (18.9)	1.8	10 [0; 10]	93 (20.1)	0	2 [0; 2]	51 (23.9)		0		5 [0; 5]	
South Australia	249 (9.11)	^1^	0	5 [0; 5]	^1^	0	0 [0; 0]	^1^		0		3 [0; 3]	
Victoria	776 (28.3)	52 (20.5)	1.1	7 [0; 7]	160 (34.6)	0	2 [0; 2]	83 (39.0)		0		5 [0; 5]	
Western Australia	248 (9.07)	^1^	0	3 [0; 3]	^1^	0	0 [0; 0]	^1^		0		3 [0; 3]	

Metric	Continuity of care facilities cohort^3^	Continuity of care – known GP			Continuity of care – usual GP								
Total number of facilities	2680	2680			2680								
Median [Q1; Q3]		9.9 [0; 18.9]			13.4 [0; 27.2]								
IQR		18.9			27.2								
Median Absolute Deviation		9.9			13.4								
Inlier-range [b_lower_; b_upper_]^2^		47.1 [0; 47.1]			68 [0; 68]								
Outliers total (% of all LTCF)		84 (2.13)			81 (2.95)								
Facility characteristics	N (%)	Outliers, N (% of total outliers)	Median	Inlier-range [b_lower_; b_upper_]^2^	Outliers, N (% of total outliers)	Median	Inlier-range [b_lower_; b_upper_]^2^						
Ownership type													
Government	230 (8.58)	29 (34.5)	14.6	85 [0; 85]	24 (29.6)	20.9	100 [0; 100]						
Not-For-Profit	1537 (57.1)	45 (53.6)	10.4	49 [0; 49]	51 (63.0)	15.8	70 [0; 70]						
Private	923 (34.3)	10 (11.9)	8.8	39 [0; 39]	6 (7.41)	10.2	51 [0; 51]						
Remoteness													
Metropolitan	1691 (62.9)	16 (19.1)	7.7	37 [0; 37]	6 (7.41)	9.2	46 [0; 46]						
Non-Metropolitan	989 (36.8)	68 (81.0)	15.1	56 [0; 56]	75 (92.6)	26.8	95 [0; 95]						
Size													
1–29	2423 (90.1)	55 (65.5)	0	70 [0; 70]	47 (58.0)	12.6	100 [0; 100]						
30–59	253 (9.44)	^1^	10.7	51 [0; 51]	31 (38.3)	13.5	72 [0; 72]						
60–89	14 (0.52)	^1^	10.1	34 [0; 34]	^1^	15.8	56 [0; 56]						
90 +	0 (0)	^1^	9.4	32 [0; 32]	^1^	11.6	45 [0; 45]						
State													
New South Wales	878 (32.6)	28 (33.3)	11.7	52 [0; 52]	33 (40.7)	16.1	67 [0; 67]						
Other^4^	104 (3.88)	^1^	10.8	37 [0; 37]	^1^	19.6	66 [0; 66]						
Queensland	465 (17.3)	14 (16.7)	9.5	44 [0; 44]	8 (9.88)	11.2	66 [0; 66]						
South Australia	243 (8.89)	8 (9.52)	11.4	48 [0; 48]	^1^	15.0	54 [0; 54]						
Victoria	762 (28.3)	27 (32.1)	9.7	49 [0; 49]	28 (34.6)	13.5	67 [0; 67]						
Western Australia	238 (8.88)	^1^	0	26 [0; 26]	^1^	3.8	38 [0; 38]						

PIP = Practice Incentives Program. GP = General practitioner; Q1 = Quartile 1; Q2 = Quartile 2; IQR = interquartile range. b_lower_ = Q1-1.5*IQR, and b_upper_ = Q3 + 1.5*IQR

1. Cell numbers are masked to prevent service home identification due to small cell numbers

2. Inlier-range bounds were defined equally to the maximal potential whisker values in box-whisker-plots

3. This cohort includes homes who had at least one resident eligible for the continuity of care analysis, that is, who entered care in 2019 and stayed in the home for at least 180 days

4. Australian Capital Territory, Northern Territory, and Tasmania were grouped due to small numbers of facilities producing small cell numbers requiring masking

### Variation in utilization of healthcare services and continuity of care by LTCF characteristics

Major differences across LTCF characteristics were in after-hours attendances, where utilization ranged from 34.9/100 residents in non-metropolitan to 74.6/100 in metropolitan LTCFs and from 49.7/100 in government-operated LTCFs to 75.5/100 in private LTCFs (Table 2, Supplemental Fig. 1), but variation was maximal across all LTCF characteristics (all *inlier-ranges* = 100 [0;100]). Utilization of urgent after-hours GP/MP attendances ranged from 6/100 residents in non-metropolitan to 40/100 in metropolitan LTCFs and variation ranged from moderate to maximal across LTCF characteristics (*inlier-range =* 36 [0;36] in non-metropolitan, *inlier-range* = 100 [0;100] in metropolitan LTCFs; Table 2, Supplemental Fig. 2). Utilization of GP/MP health assessments ranged from 37.2/100 residents in non-metropolitan to 43.1/100 in metropolitan LTCFs and from 38.9/100 in not-for-profit LTCFs to 45.6/100 in private LTCFs, and variation was maximal across all LTCF characteristics (all *inlier-ranges* = 100 [0;100]; Table 2; Fig. 1). The utilization of GP/MP management plans ranged from 43.5/100 residents in non-metropolitan to 62.9/100 in metropolitan LTCFs and from 42.9/100 in government to 63.3/100 in private LTCFs, with maximal variation across all LTCF characteristics (*inlier-ranges* = 100 [0;100]; Table 2, Supplemental Fig. 3).

Podiatry services utilization ranged from 17.7/100 residents in government-operated LTCFs to 36.5/100 in private LTCFs and from 19.1/100 in non-metropolitan to 41.8/100 in metropolitan LTCFs, but variation was maximal across LTCF characteristics (*inlier-ranges* = 100 [0;100]; Table 2, Supplemental Fig. 4). Optometric services utilization ranged from 25.2/100 residents in non-metropolitan to 51/100 in metropolitan LTCFs and from 30.6/100 in government-operated LTCFs to 53.2/100 in private LTCFs, and variation was maximal across LTCF characteristics (*inlier-ranges* = 100 [0;100]) except for non-metropolitan LTCFs (*inlier-range* = 74 [0;74]; Table 2, Supplemental Fig. 5). Utilization of comprehensive medication reviews was moderate in all LTCF characteristics, and variation was high across LTCF characteristics (*inlier-range* = 100 [0;100] in LTCFs with 1–29 residents, *inlier-range =* 74 [0;74] in LTCFs with 90 + residents; Table 2, Supplemental Fig. 6). Variation in utilization of comprehensive medication reviews ranged from 26.9/100 residents in government operated LTCFs to 34.1/100 in private LTCFs and from 29.6/100 in non-metropolitan LTCFs to 34.7/100 in metropolitan LTCFs.

Utilization of geriatric specialist attendances was low, and variation was low to moderate (Table 2, Supplemental Fig. 7). Utilization of multimorbidity specialist attendances was low, with moderate variation across LTCF characteristics (Table 2, Supplemental Fig. 8).

Across all types of LTCFs, utilization of GP/MP general attendances was high and variation minimal to low (Table 2, Supplemental Fig. 10). Utilization of nurse practitioner attendances (Table 2; Fig. 3), GP/MP attendances associated with PIP (Table 2, Supplemental Fig. 11), pain specialists (Table 2, Supplemental Fig. 12), and all mental health services were very low (Table 2, Supplemental Figs. 13–16), and variation was low across all LTCF characteristics.

Continuity of care with a known GP/MP was low across LTCF subgroups, ranging from 15.1/100 in non-metropolitan LTCFs to 7.7/100 residents in metropolitan LTCFs and from 0/100 residents in LTCFs with 1–29 residents to 10.7/100 in LTCFs with 30–59 residents, and variation was moderate to high across LTCF characteristics (*inlier-range =* 70 [0;70] in LTCFs with 1–29 residents, inlier-range = 32 [0;32] in LTCFs with 90 + residents; Table 2, Supplemental Fig. 9). Continuity of care with the usual GP/MP was low across LTCF subgroups and variation was low across LTCF characteristics (Table 2; Fig. 2).

### Characteristics of healthcare service utilization and continuity of care LTCF outliers

Outliers identified had a higher prevalence of service utilization, except regarding general GP/MP attendances. The number of outlier LTCFs for each of the 12 healthcare services ranged from 12 in comprehensive medication reviews (individual characteristics suppressed to prevent identifiability) to 463 in nurse practitioner attendances (59.6% [276/463] not-for-profit, 71.9% [333/463] metropolitan, 54.2% [146/463] sized 60 + residents; Table 3). There were 81 outlier LTCFs for the continuity of care - usual GP category (63% [51/81] not-for-profit, 92.6% [75/81] non-metropolitan, 58% [47/81] sized 1–29 residents, Table 3), and 84 outlier LTCFs for the known GP category (53.6% [45/84] not-for-profit, 65.5% [55/84] sized 1–29 residents; Table 3).

## Discussion

This national investigation into the variation of government subsidized primary and specialist healthcare service utilization and continuity of care experienced by individuals living in LTCFs has identified high variation across most of the services examined– in the public health context, this variation can be considered *unwarranted* [8, 24], as it is likely not explained by care or health needs of individuals. Whereas utilization levels differed by LTCF characteristics, variation in utilization did not. Specifically, we identified substantial variation in service utilization for after-hours and urgent after-hours GP attendances, health assessments, management plans, podiatry attendances, optometric attendances, comprehensive medication reviews, and continuity of care. Of all LTCFs outliers, most were high outliers with a higher prevalence of service utilization, except for GP/MP attendances which had a generally high utilization across all facility characteristics.

Previous work has posited that unwarranted variation often stems from uncertainty about best practices [25]. Our study indicates that such uncertainty surrounds the delivery of GP management plans and health assessments in Australian LTCFs. Although both services are recommended for all older people living in LTCFs, we observed only moderate levels of utilization and high variation. The results suggest that their utilization may be more strongly influenced by provider preferences and subjective beliefs about their effectiveness than by adherence to clinical guidelines, suggesting unwarranted variation.

This study has identified substantial variation between LTCFs regarding primary and specialist service utilization, and it is likely that system-level factors (e.g., related to workforce, funding incentives, and LTCF care models) are partly responsible for this variation [8, 24]. Variation in utilization of specialist attendances is likely due to access limitations– most specialist providers live and work in metropolitan areas [26]. Similarly, the very low use and high variation in nurse practitioner service may be related to national workforce limitations (i.e., few nurse practitioners nationally, most work in acute care facilities) [27]. Additionally, it is plausible that provider differences other than those we examined may explain some of the variation identified; for example, the implementation of different models of care including salaried providers or partnerships with providers salaried by other institutions. Variation in continuity of GP care is plausibly related to access (i.e., the distances people move in order to enter a LTCF), or differences in how LTCFs manage healthcare (i.e., many LTCFs have arrangements with specific GPs who become the new GP for residents upon entry). While Standards [28] dictate that LTCF residents must be able to choose their GP (i.e., LTCFs cannot insist residents see a particular GP), the usual or known GP may not be nearby. Additionally, previous work has suggested that characteristics of the healthcare providers, including their expert opinion or preferences, may explain variation in such instances [29]. Some healthcare providers may be more likely to prescribe a particular treatment (or treatment approach) based on their beliefs about the utility/benefit of the treatment, though this is inherently subjective [29]. While these factors may explain some of the observed variation, these findings demonstrate the need to mitigate unwarranted variation. Indeed, our findings support calls to identify unwarranted variation in healthcare delivery so that improvement strategies can target the local economic, cultural, and situational needs of people receiving care [10].

A significant body of international evidence has identified that LTCF characteristics are associated with resident outcomes [30–36]. Therefore, it is reasonable that healthcare service utilization and its variation would differ based on these characteristics. The Australian Government Royal Commission into Aged Care Quality and Safety reported that LTCF size, ownership type, and location were associated with certain outcomes [33–35]. Consistent with the Royal Commission reporting, recent work has found that higher (i.e., better) LTCF Star Ratings were associated with smaller and government-operated LTCFs [31]. Application of recommendations for care may be associated with facility characteristics. For example, government owned facilities may be more likely to systematically implement standards of care, leading to higher utilization with lower variation for some services, as observed for GP/MP attendances, where government-owned facilities showed less variation in utilization of urgent after-hours attendances than not-for-profit or privately owned LTCFs. Similarly, variation in continuity of care with a known GP was higher among smaller, government-owned, non-metropolitan LTCFs; notably, the observed variation for these facilities was still substantial. Additionally, previous work has identified comprehensive medication reviews upon entry to LTCFs as an underutilized service– our findings extend this work by showing high variation in the utilization of this service [17].

While LTCF characteristics have been associated with outcomes for residents, much of the variation identified did not notably differ by facility characteristics. While our findings evaluate the nature of the association between LTCF characteristics and variation in service utilization, the facility characteristics evaluated herein may be associated with other organizational and economic factors. For example, previous work has shown that economic factors and characteristics of healthcare providers (e.g., job resources, job demands) are associated with quality of care [37, 38]. Additionally, models of care may be tied to LTCF characteristics. Whereas large LTCFs are common in Australia, other models of residential care are increasingly common around the world, though these should be implemented and evaluated in the context of the individuals for whom they are most appropriate. The *clustered domestic* model is characterized by a physical design that prioritizes a more home-like environment for residents and is suitable for those with relatively low disability and frailty, and has been shown to be associated with better outcomes for residents (e.g., fewer hospitalizations and higher quality of life) [39]. The *Eden Alternative* has been demonstrated in Australia and New Zealand to be a suitable model of residential aged care for people with dementia, and may result in more positive outcomes and quality of care [40]. Importantly, different models of care will necessarily utilize MBS healthcare reimbursement differently, making this measure of utilization an incomplete reflection of quality of care. A more precise description of models of care, not only in terms of provider services but also regarding facility structure may facilitate future research to evaluate drivers of unwarranted variation in service utilization.

Overall healthcare service utilization varied substantially nationally, with both positive and negative outliers observed in specific services. Being an outlier may be related to differences in clinical governance, staffing arrangements, and service delivery models. For example, higher utilization of nurse practitioners may reflect that the LTCFs have embedded nurse practitioners or maintain formal agreements with allied health providers. In contrast, low utilization of general GP/MP attendances may reflect that some LTCFs lack regular visiting medical practitioners—a problem that has also been highlighted in the Final Report of the Royal Commission into Aged Care Quality and Safety [4].

Continuity of care, in particular, showed substantial variation, with the most positive outlier LTCFs being government-run and located in non-metropolitan areas. This may be explained by stronger existing ties between residents and local GPs in these settings, fewer available providers, and more stable relationships [41, 42]. While these characteristics might improve continuity, they simultaneously may limit access generally, if broader primary care capacity is lacking. This interplay between continuity and ownership type points to a complex system in which outlier status is not only a function of availability but also how services are organized and accessed.

Importantly, the interdependence between services (e.g., high after-hours service use possibly compensating for low continuity) suggests that some patterns are symptomatic of broader care models—such as reactive versus preventive approaches to care—rather than anomalies. Our results suggest that residents in private LTCFs are more likely to have care utilization patterns indicating reactive care than residents in government or not-for-profit LTCFs (Table 3; Fig. 2, Supplemental Fig. 1, Supplemental Fig. 2). This warrants further study, but our findings underscore the need to explore facility- and provider-level care models, provider relationships, and geographical context as potential levers for improving consistency in access.

Our study has several limitations, most notably that the dataset includes only government subsidized healthcare services. Services accessed privately through aged care providers or state funded programs are not captured and so the utilization of such services may be underestimated (e.g., optometric services) [43]. Our examination of variation and contributors to LTCF outliers was limited; it is plausible that factors such as models of care, workforce, and economic factors drive this variation. We did not include certain clinically important covariates such as activities of daily living or cognitive impairment variables in our risk adjustment to maintain comparability with other notable approaches. This omission may have resulted in residual confounding, and unmeasured differences in residents’ functional status and other health conditions might partially account for the variability observed. Relatedly, our analysis does not allow inferences about appropriateness of service utilization on the level of individuals or individual facilities. Strengths of this work include representativeness of the population studied due to the use of national population data and the comprehensive investigation into patterns of primary and specialist healthcare services accessed by older people in Australia, the use of selected specialist services of particular interest to older people in LTCFs, and continuity of GP care. Our findings are also likely generalizable to countries with universal health and aged care sectors and comparable populations (e.g., Canada, United Kingdom, New Zealand) [44]. Additionally, the information used for this study is obtained from several long-standing data collections, including assessments undertaken by clinically trained assessors, used together to improve the internal validity of variables included in our analyses (e.g., health conditions).

## Conclusions

Our findings demonstrate plausibly unwarranted variation in the utilization of a variety of healthcare services in LTCFs. We do not identify a trend within these differences in utilization, suggesting that efforts to improve the quality and sustainability of care would benefit from the overall system-level strategies and standardized uptake of guidelines for utilization of primary and specialist care services. Following recent calls for identification and implementation of sustainable strategies for ensuring long-term access to quality care, [45] this study provides evidence that some services are provided inconsistently and national strategies are needed to ensure equitable and evidence-based access to healthcare in LTCFs.

## Supplementary Information

Supplementary Material 1

## Data Availability

The data underlying this report are not available for sharing because of restrictions imposed by the ethics and original data custodian approval.

## Abbreviations

LTCF: Long-term care facility
GP: General practitioner
MP: Medical practitioner
PBS: Pharmaceutical benefits scheme
MBS: Medicare benefits schedule
NDI: National death index
ROSA: Registry of senior australians
DVA: Department of Veterans’ Affairs
IQR: Interquartile-range
PIP: Practice incentives program
