# Supplementary material for: Variation in healthcare services utilization and continuity of care in long-term care facilities: a cross-sectional study
Source: BMC Health Serv Res. 2025 Sep 29;25:1216. doi: 10.1186/s12913-025-13321-4 (PMC12482496; doi:10.1186/s12913-025-13321-4)
Supplement: Supplementary file 1 — Supplementary Material 1 [file 12913_2025_13321_MOESM1_ESM.docx]

**Supplemental Materials**

**Supplemental Tables**

**Supplemental Table 1. Exposures of Interest: Healthcare Services Coding**

| Health Care Services Exposures | | | | |
| --- | --- | --- | --- | --- |
| MBS Ascertained | MBS Groups | MBS Group Description | MBS items |  |
| General Attendances | | | | |
| GP/Medical practitioner attendances | A01  A02  A35 | GP attendances  Non-referred attendance to medical practitioner  Medical services at residential aged care facilities | 3, 4, 23, 24, 36, 37, 44, 47  52, 53, 54 ,57, 58, 59, 60, 65  20, 35, 43, 51, 92, 93, 95, 96, 183, 188, 202, 212, 90020, 90035, 90043, 90051, 90092, 90093, 90095, 90096,  90183, 90188, 90202, 90212 |  |
| After-hours attendances | A22  A23 | GP after-hours attendance  Non-referred after-hours attendance with medical practitioners | 5000, 5003, 5010, 5020, 5023, 5028, 5040, 5043, 5049, 5060, 5063, 5067  5200, 5203, 5207, 5208, 5220, 5223, 5227, 5228, 5260, 5263, 5265, 5267 |  |
| Urgent attendance after-hours | A11 | Urgent GP/Medical practitioner attendance after-hours | 585, 588, 591, 594, 597, 598, 599, 600 |  |
| Nurse practitioners attendances | M14 | Nurse practitioners | 82200, 82205, 82210, 82215, 82220, 82221, 82222, 82223, 82224, 82225 |  |
| Health Assessments / Management Plans | | | | |
| Health assessments | A14 | GP/Medical practitioner health assessments | 224, 225, 226, 227, 701, 703, 705, 707 |  |
| Management plans | A15 | GP/Medical practitioner management plan attendances/team care arrangements and multidisciplinary care plans | 229, 230, 231, 232, 233, 235, 236, 237, 238, 239, 240, 243, 244, 721, 723, 729, 731, 732, 735, 739, 743, 747, 750, 758, 871, 872 |  |
| GP/ Medical practitioner attendance associated with PIP/non-referred attendance associated with PIP | A18/19 | GP/Medical practitioner attendance associated with PIP/non-referred attendance associated with PIP | 251, 252, 253, 254, 255, 256, 257, 259, 260, 261, 262, 263, 265, 266, 268, 269, 270, 271, 2497, 2501, 2503, 2504, 2506, 2507, 2509, 2517, 2518, 2521, 2522, 2525, 2526, 2546, 2547, 2552, 2553, 2558, 2559, 2598, 2600, 2603, 2606, 2610, 2613, 2616, 2620, 2622, 2624, 2631, 2633, 2635, 2664, 2666, 2668, 2673, 2675, 2677 |  |
| Allied Health Services | | | | |
| Allied health service part of CDMP | M03 | Allied health component of chronic disease management plan (GP management plan)  Podiatry services | 10951, 10952, 10953, 10954, 10956, 10958, 10960, 10962, 10964, 10966, 10968, 10970  10962 |  |
| Optometric services | A10 | Optometric services | 10905, 10907, 10911, 10912, 10913, 10915, 10916, 10918, 10922, 10923, 10924, 10925, 10926, 10927, 10928, 10929, 10930, 10931, 10932, 10933, 10940, 10941, 10942, 10943, 10944, 10945, 10946, 10947, 10948 |  |
| Comprehensive medication review | A17 | Domiciliary and residential medication management reviews | 245, 249, 900, 903 |  |
| Selected Specialist Attendances | | | |  |
| Pain medicine attendances | A24 | Pain medicine attendances | 2799, 2801, 2806, 2814, 2820, 2824, 2832, 2840, 2946, 2949, 2954, 2958, 2972, 2974, 2978, 2984, 2988, 2996, 3000 |  |
| Geriatric medicine | A28 | Geriatric medicine attendances | 141, 143, 145, 147, 149 |  |
| Multimorbidity medicine attendances | A04 | Consultant physician attendances to which no other item applies, patient with at least 2 morbidities (multiple morbidities) | 132, 133 |  |
| Mental Health Services | | | |  |
| Psychiatry attendances | A08 | Consultant psychiatric services | 288, 291, 293, 296, 297, 299, 300, 302, 304, 306, 308, 310, 312, 314, 316, 318, 319, 320, 322, 324, 326, 328, 330, 332, 334, 336, 338, 342, 344, 346, 348, 350, 352, 353, 355, 356, 357, 358, 359, 361, 364, 366, 367, 369, 370 |  |
| Psychological therapy | M06 | Psychological therapy services | 80000, 80001, 80005, 80010, 80011, 80015, 80020, 80021 |  |
| Focussed psychological strategies | M07 | Focussed psychological strategies | 941, 942, 2721, 2723, 2725, 2727, 80100, 80101, 80105, 80110, 80111, 80115, 80120, 80121, 80125, 80126, 80130, 80135, 80136, 80140, 80145, 80146, 80150, 80151, 80155, 80160, 80161, 80165, 80170, 80171 |  |
| GP mental health | A20 | GP mental health treatment | 272, 276, 277, 279, 281, 282, 2700, 2701, 2702, 2712, 2713, 2715, 2717, 2719 |  |
| Derived Exposure | | | | |
| Services included in the identification of continuity of primary care categories | | | | |
| GP/Medical practitioner attendances  GP/Medical practitioner after-hours attendances  Urgent GP/Medical practitioner attendance after-hours  Health assessments  Management plans | A01  A02  A35    A22  A23  A11  A14  A15 | GP attendance  Non-referred attendance to medical practitioner  Medical services at residential aged care facilities    GP after-hours attendance  Non-referred after-hours attendance with medical practitioners  Urgent GP after-hours attendance  GP/Medical practitioner health assessments  GP management plan attendances/team care arrangements and multidisciplinary care plans | 3, 4, 23, 24, 36, 37, 44, 47  52, 53, 54 ,57, 58, 59, 60, 65  20, 35, 43, 51, 92, 93, 95, 96, 183, 188, 202, 212, 90020, 90035, 90043, 90051, 90092, 90093, 90095, 90096, 90183, 90188, 90202, 90212  5000, 5003, 5010, 5020, 5023, 5028, 5040, 5043, 5049, 5060, 5063, 5067  5200, 5203, 5207, 5208, 5220, 5223, 5227, 5228, 5260, 5263, 5265, 5267  585, 588, 591, 594, 597, 598, 599, 600  224, 225, 226, 227, 701, 703, 705, 707  229, 230, 231, 232, 233, 235, 236, 237, 238, 239, 240, 243, 244, 721, 723, 729, 731, 732, 735, 739, 743, 747, 750, 758, 871, 872 |  |

Abbreviations: GP=General Practitioner, PIP=Practice Incentives Program, CDMP=Chronic Disease Management Plan.

**Supplemental Figures**

**Supplemental Figure 1. Combined strip chart and box-whisker plots visualising the adjusted prevalence of utilisation of GP/MP After-hours attendances in 100 residents of residential aged care homes in 2019 overall and by home characteristics.**


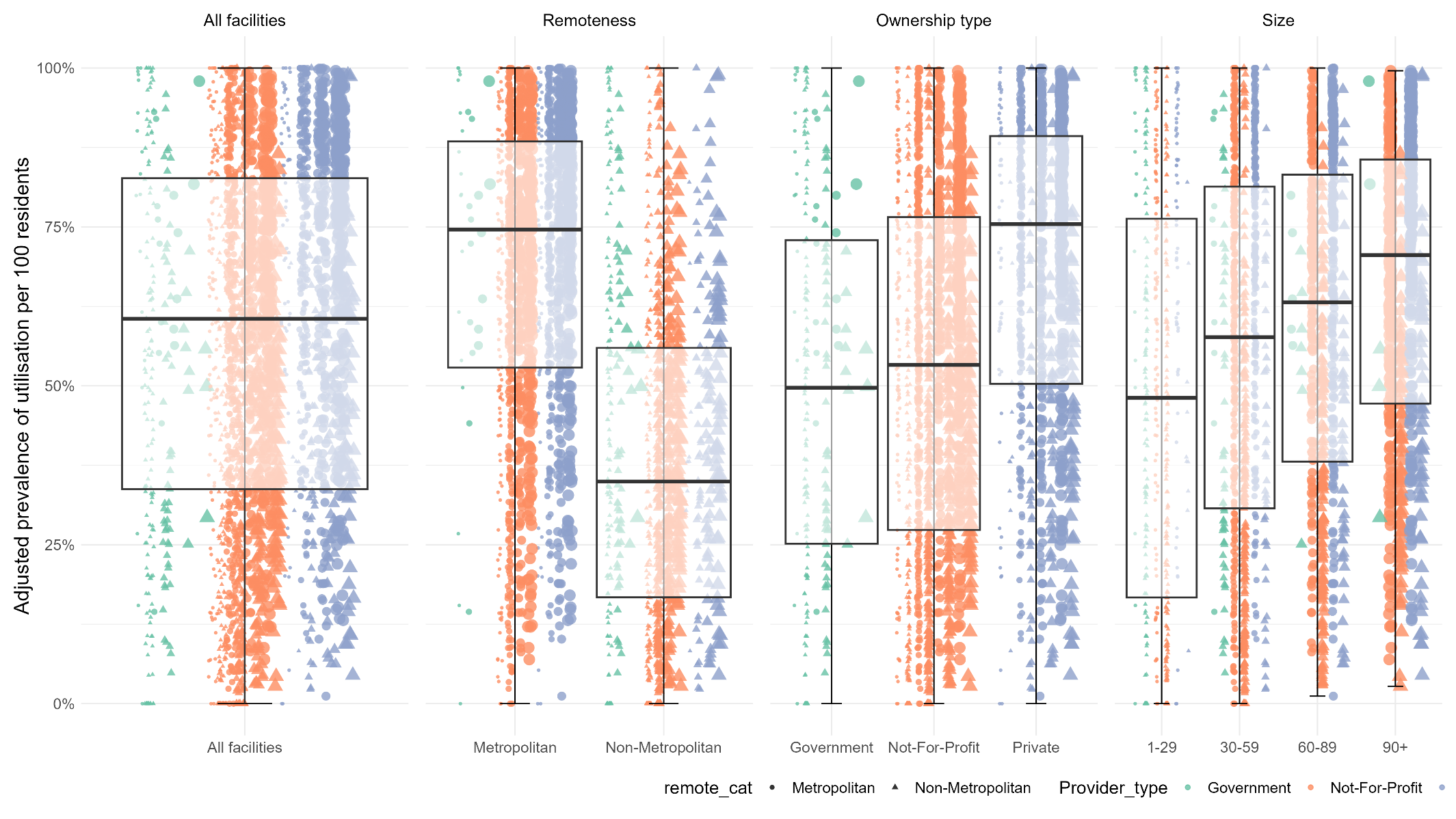

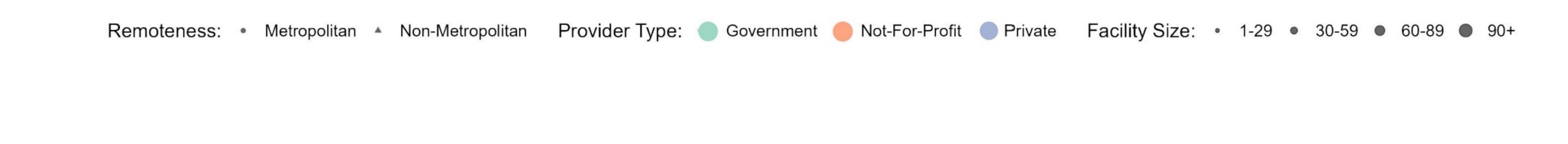


Note. N=2733. On an individual level, utilisation of a service was measures binary with at least one utilisation versus none. The adjusted prevalence of utilisation in 100 residents can thus be understood similar to a percentage of residents who got the service, adjusted by resident-mix in terms of age, sex, and comorbidities. Each data point represents one service home. The shape of a point represents service home remoteness (dots=metropolitan, triangles=non-metropolitan). The size of a data point represents the service home size. The colour represents the ownership type. Data points classified as outliers are represented with grey dots mirroring their utilisation level above or below the whiskers.

**Supplemental Figure 2. Combined strip chart and box-whisker plots visualising the adjusted prevalence of utilisation of Urgent GP/MP After-hours attendances in 100 residents of residential aged care homes in 2019 overall and by home characteristics.**


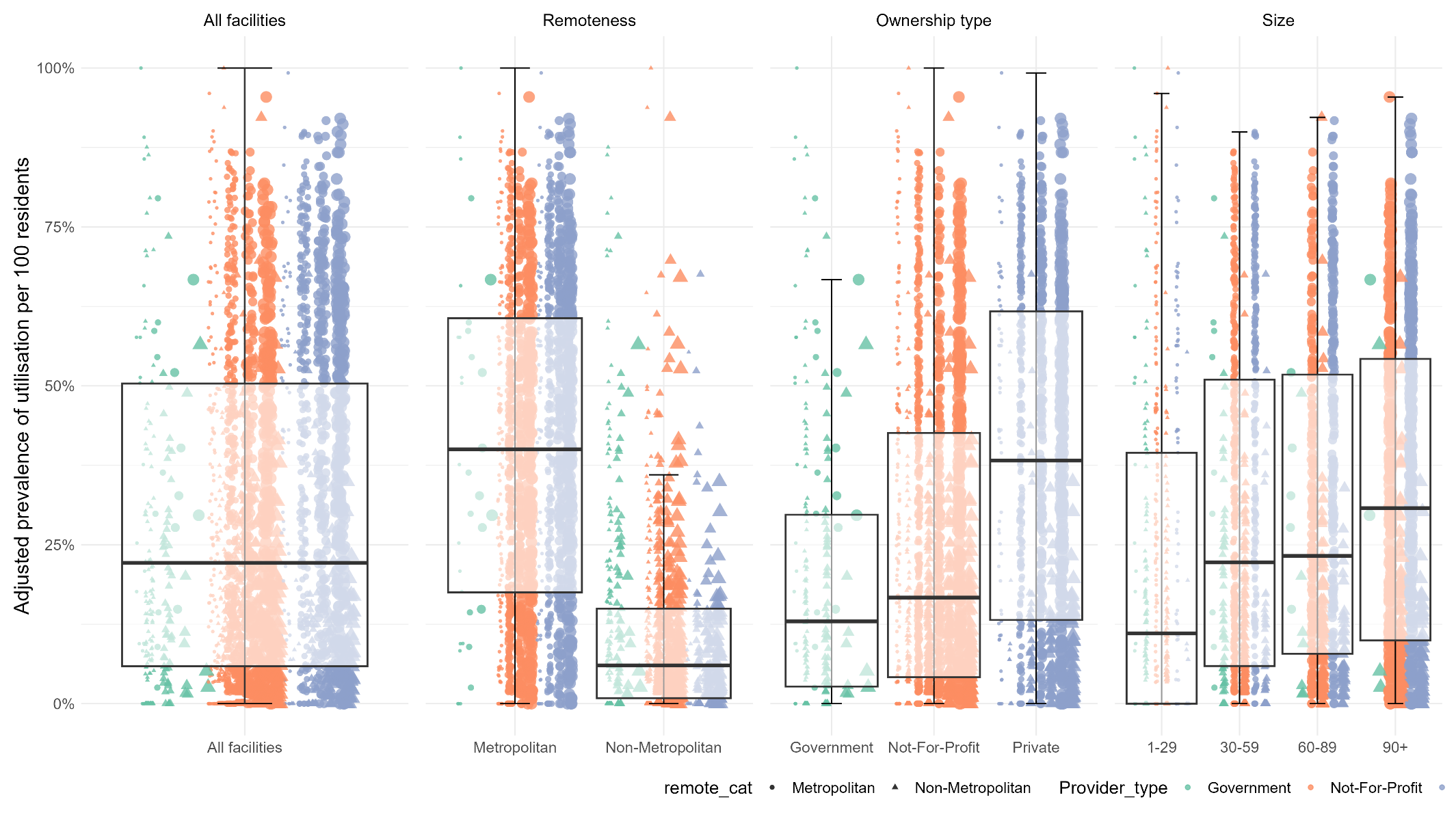

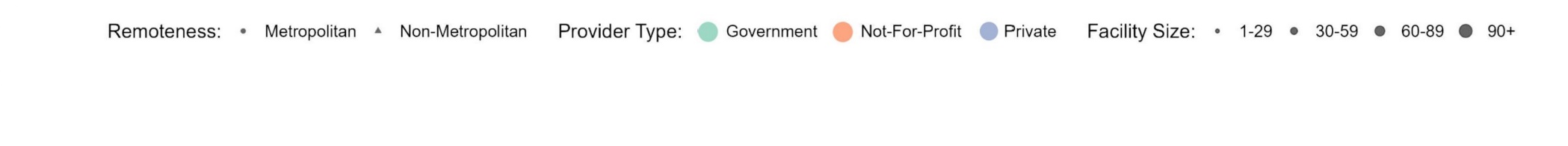


Note. N=2733. On an individual level, utilisation of a service was measures binary with at least one utilisation versus none. The adjusted prevalence of utilisation in 100 residents can thus be understood similar to a percentage of residents who got the service, adjusted by resident-mix in terms of age, sex, and comorbidities. Each data point represents one service home. The shape of a point represents service home remoteness (dots=metropolitan, triangles=non-metropolitan). The size of a data point represents the service home size. The colour represents the ownership type. Data points classified as outliers are represented with grey dots mirroring their utilisation level above or below the whiskers.

**Supplemental Figure 3. Combined strip chart and box-whisker plots visualising the adjusted prevalence of utilisation of GP/MP Management Plans in 100 residents of residential aged care homes in 2019 overall and by home characteristics.**


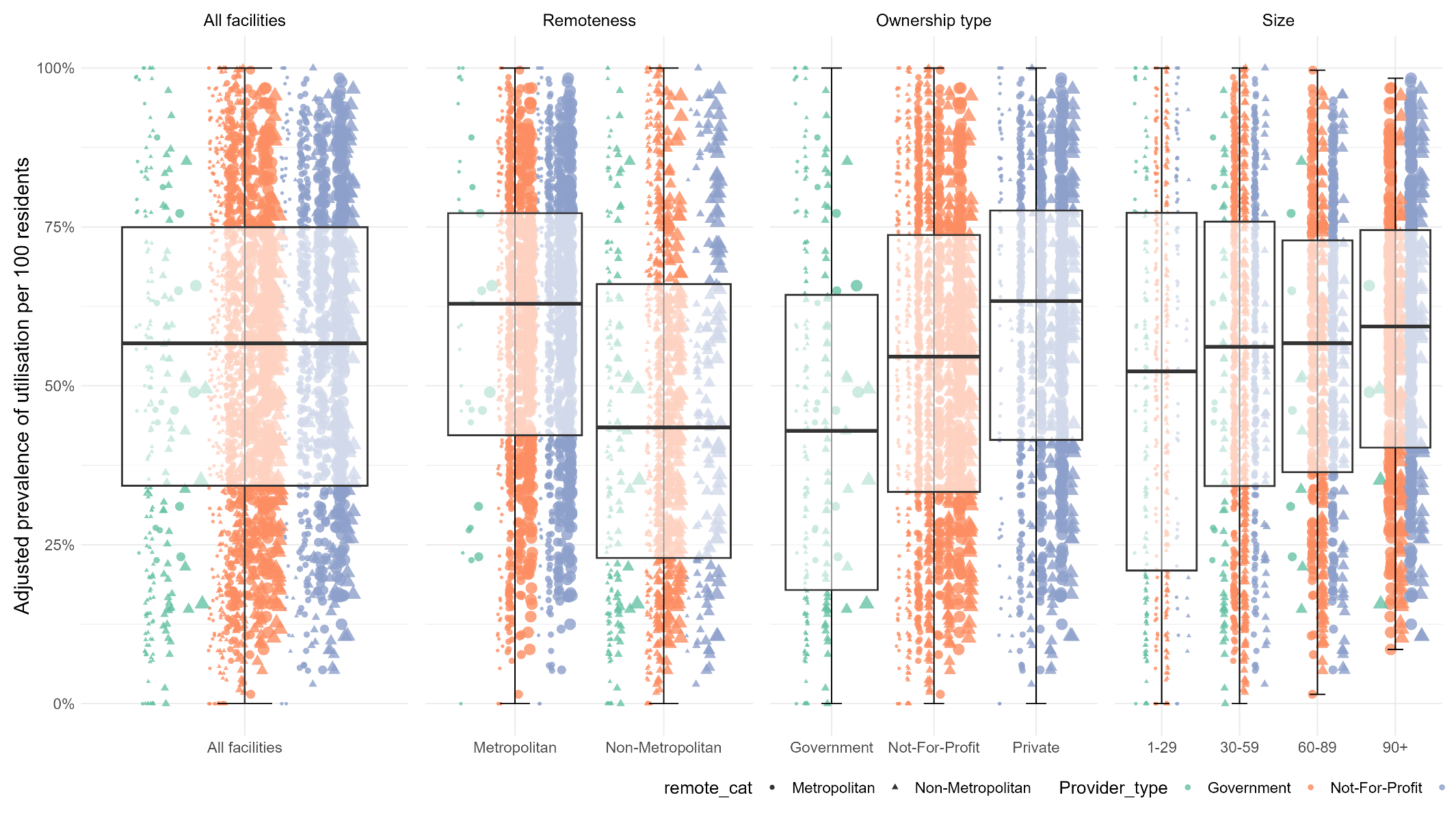

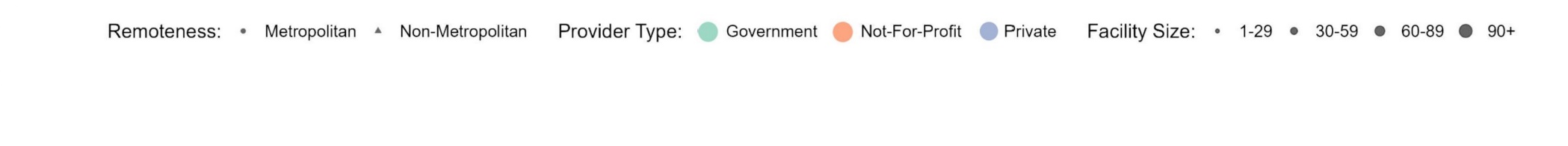

Note. N=2733. On an individual level, utilisation of a service was measures binary with at least one utilisation versus none. The adjusted prevalence of utilisation in 100 residents can thus be understood similar to a percentage of residents who got the service, adjusted by resident-mix in terms of age, sex, and comorbidities. Each data point represents one service home. The shape of a point represents service home remoteness (dots=metropolitan, triangles=non-metropolitan). The size of a data point represents the service home size. The colour represents the ownership type. Data points classified as outliers are represented with grey dots mirroring their utilisation level above or below the whiskers.

**Supplemental Figure 4. Combined strip chart and box-whisker plots visualising the adjusted prevalence of utilisation of Allied Health – Podiatry in 100 residents of residential aged care homes in 2019 overall and by home characteristics.**


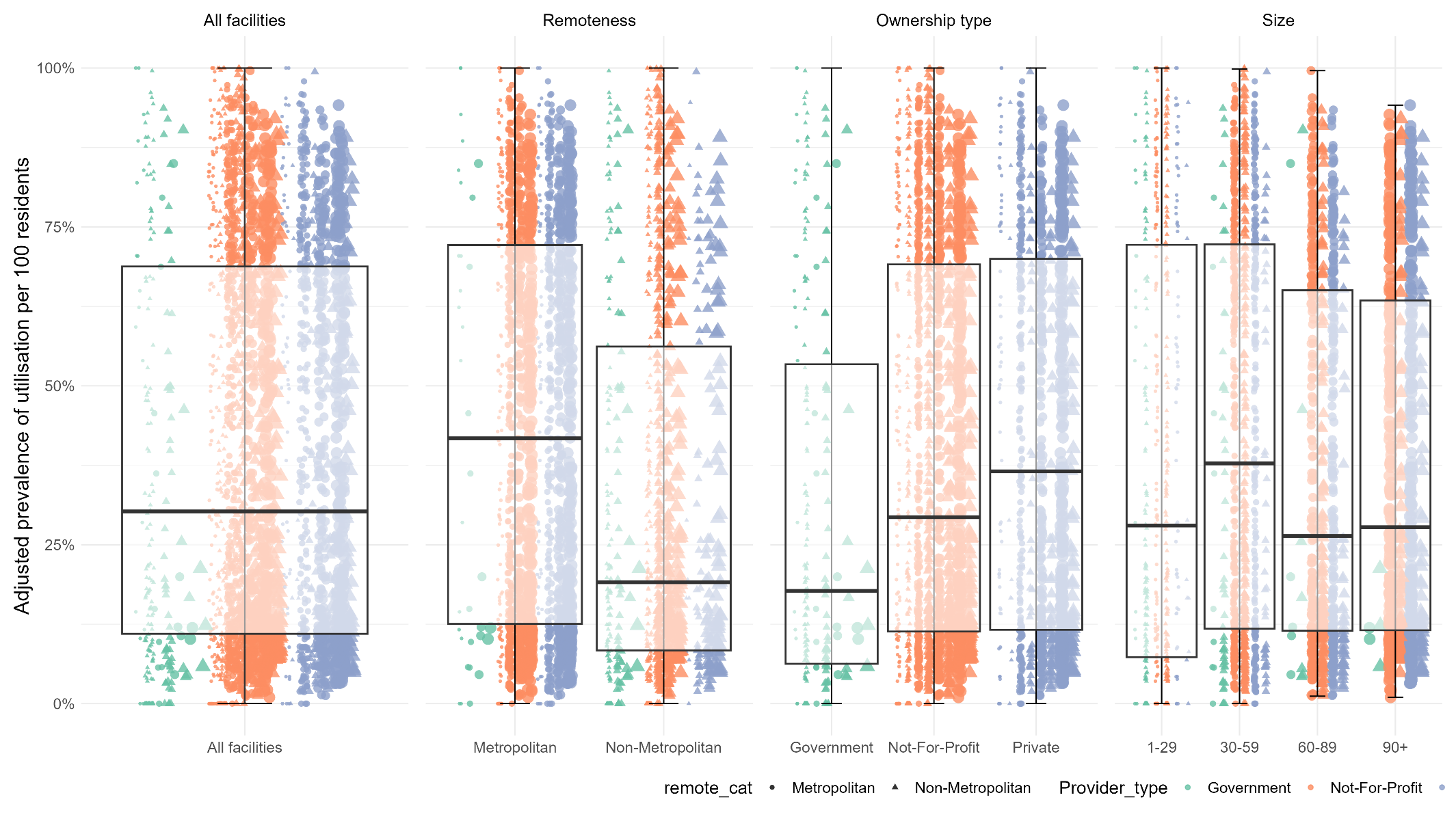


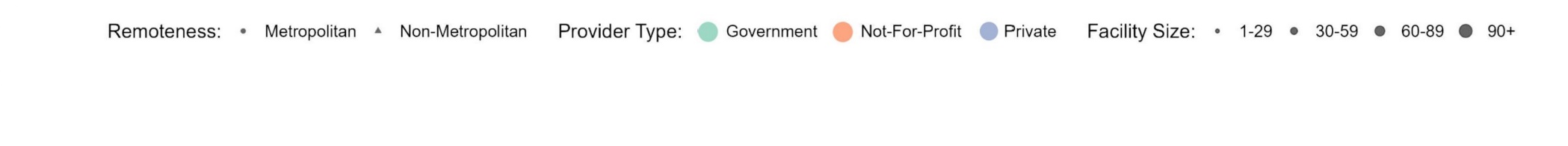


Note. N=2733. On an individual level, utilisation of a service was measures binary with at least one utilisation versus none. The adjusted prevalence of utilisation in 100 residents can thus be understood similar to a percentage of residents who got the service, adjusted by resident-mix in terms of age, sex, and comorbidities. Each data point represents one service home. The shape of a point represents service home remoteness (dots=metropolitan, triangles=non-metropolitan). The size of a data point represents the service home size. The colour represents the ownership type. Data points classified as outliers are represented with grey dots mirroring their utilisation level above or below the whiskers.

**Supplemental Figure 5. Combined strip chart and box-whisker plots visualising the adjusted prevalence of utilisation of Allied Health – Optometric Services in 100 residents of residential aged care homes in 2019 overall and by home characteristics.**


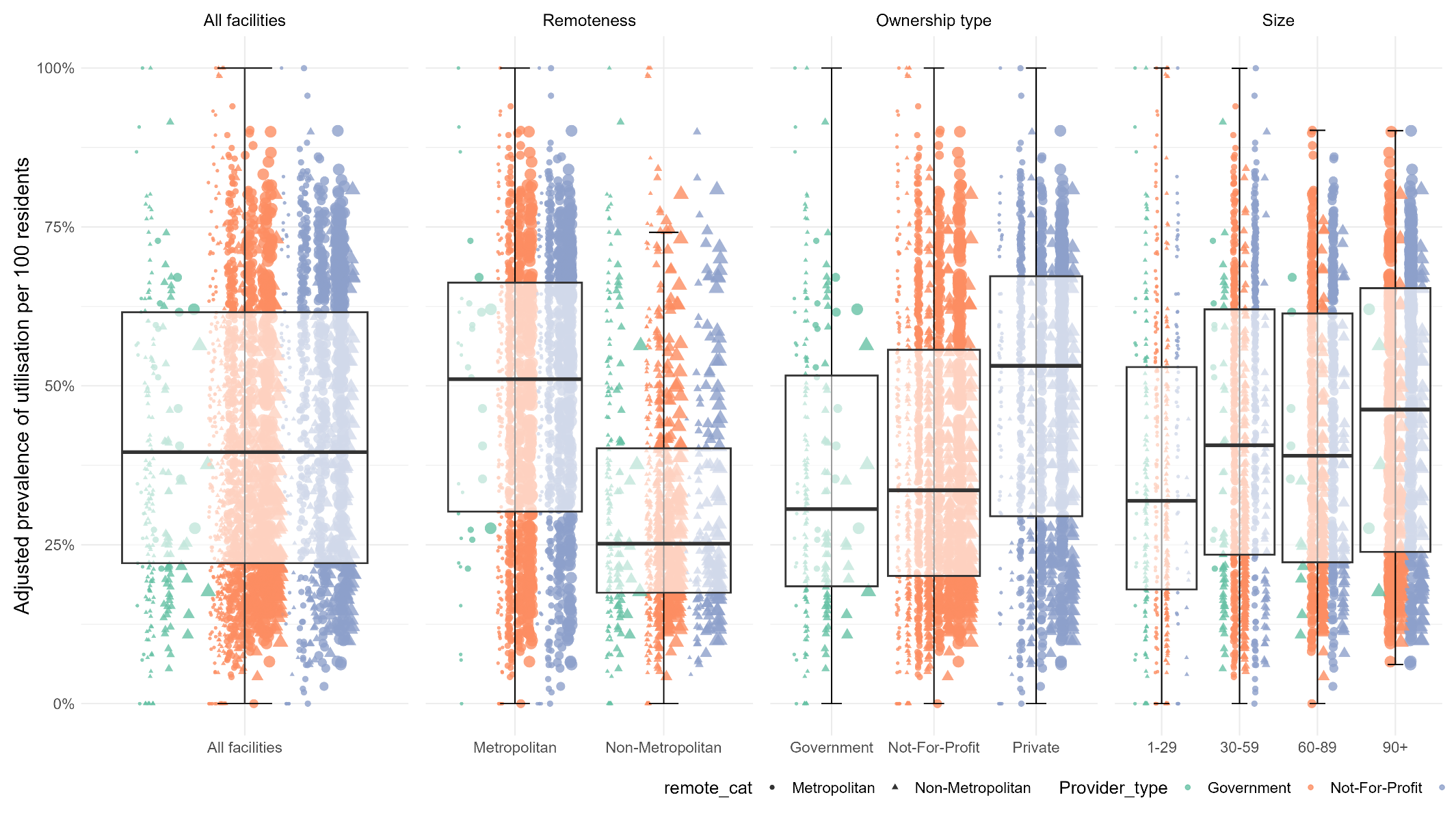


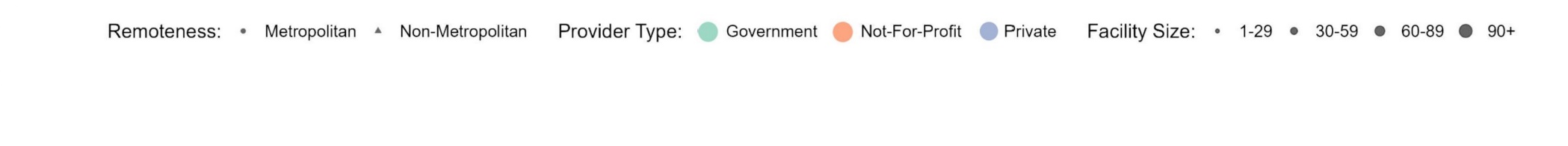


Note. N=2733. On an individual level, utilisation of a service was measures binary with at least one utilisation versus none. The adjusted prevalence of utilisation in 100 residents can thus be understood similar to a percentage of residents who got the service, adjusted by resident-mix in terms of age, sex, and comorbidities. Each data point represents one service home. The shape of a point represents service home remoteness (dots=metropolitan, triangles=non-metropolitan). The size of a data point represents the service home size. The colour represents the ownership type. Data points classified as outliers are represented with grey dots mirroring their utilisation level above or below the whiskers.

**Supplemental Figure 6. Combined strip chart and box-whisker plots visualising the adjusted prevalence of utilisation of Comprehensive Medication Reviews in 100 residents of residential aged care homes in 2019 overall and by home characteristics.**


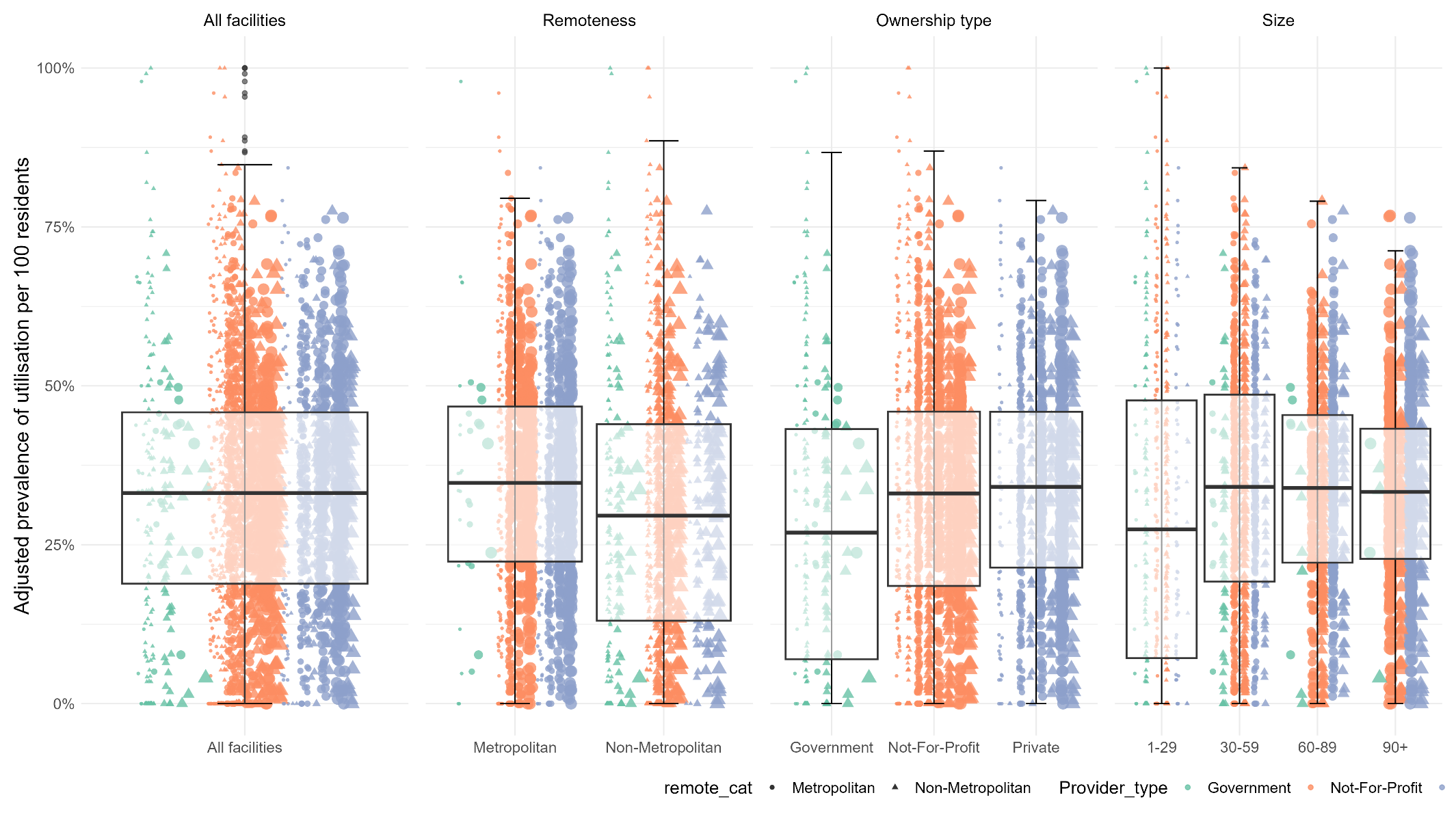


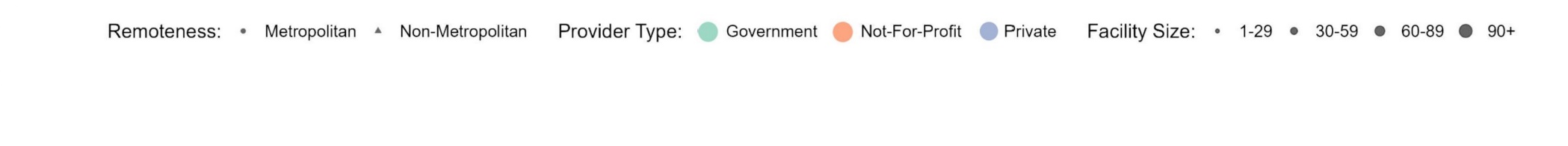


Note. N=2733. On an individual level, utilisation of a service was measures binary with at least one utilisation versus none. The adjusted prevalence of utilisation in 100 residents can thus be understood similar to a percentage of residents who got the service, adjusted by resident-mix in terms of age, sex, and comorbidities. Each data point represents one service home. The shape of a point represents service home remoteness (dots=metropolitan, triangles=non-metropolitan). The size of a data point represents the service home size. The colour represents the ownership type. Data points classified as outliers are represented with grey dots mirroring their utilisation level above or below the whiskers.

**Supplemental Figure 7. Combined strip chart and box-whisker plots visualising the adjusted prevalence of utilisation of Geriatric Medicine Specialists in 100 residents of residential aged care homes in 2019 overall and by home characteristics.**


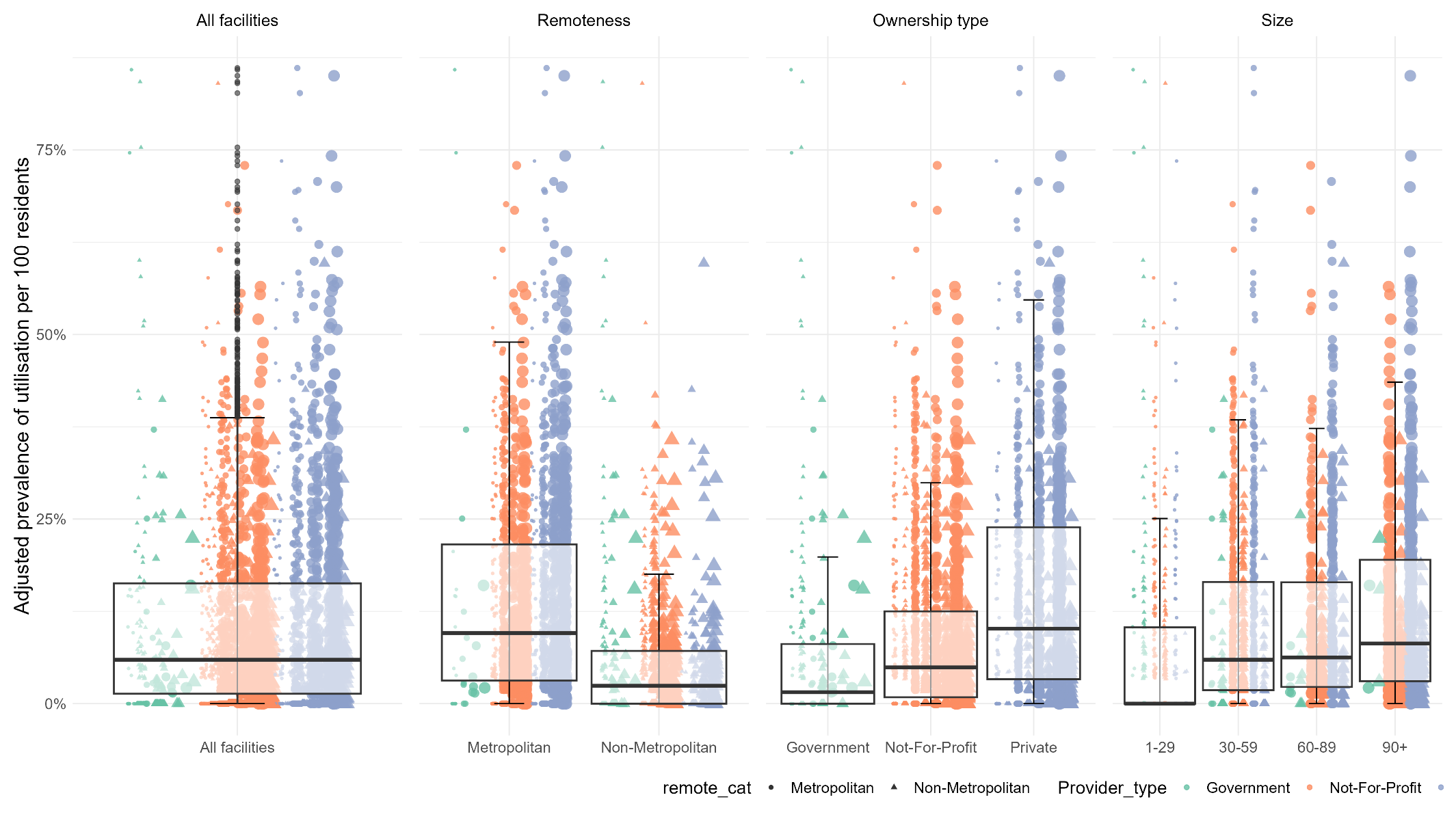

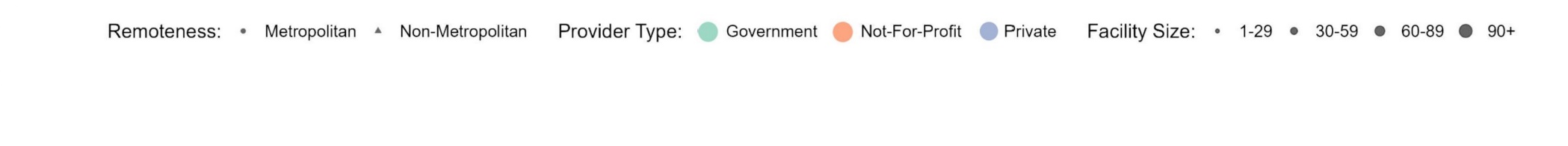


Note. N=2733. On an individual level, utilisation of a service was measures binary with at least one utilisation versus none. The adjusted prevalence of utilisation in 100 residents can thus be understood similar to a percentage of residents who got the service, adjusted by resident-mix in terms of age, sex, and comorbidities. Each data point represents one service home. The shape of a point represents service home remoteness (dots=metropolitan, triangles=non-metropolitan). The size of a data point represents the service home size. The colour represents the ownership type. Data points classified as outliers are represented with grey dots mirroring their utilisation level above or below the whiskers.

**Supplemental Figure 8. Combined strip chart and box-whisker plots visualising the adjusted prevalence of utilisation of Multimorbidity Medicine Specialists in 100 residents of residential aged care homes in 2019 overall and by home characteristics.**


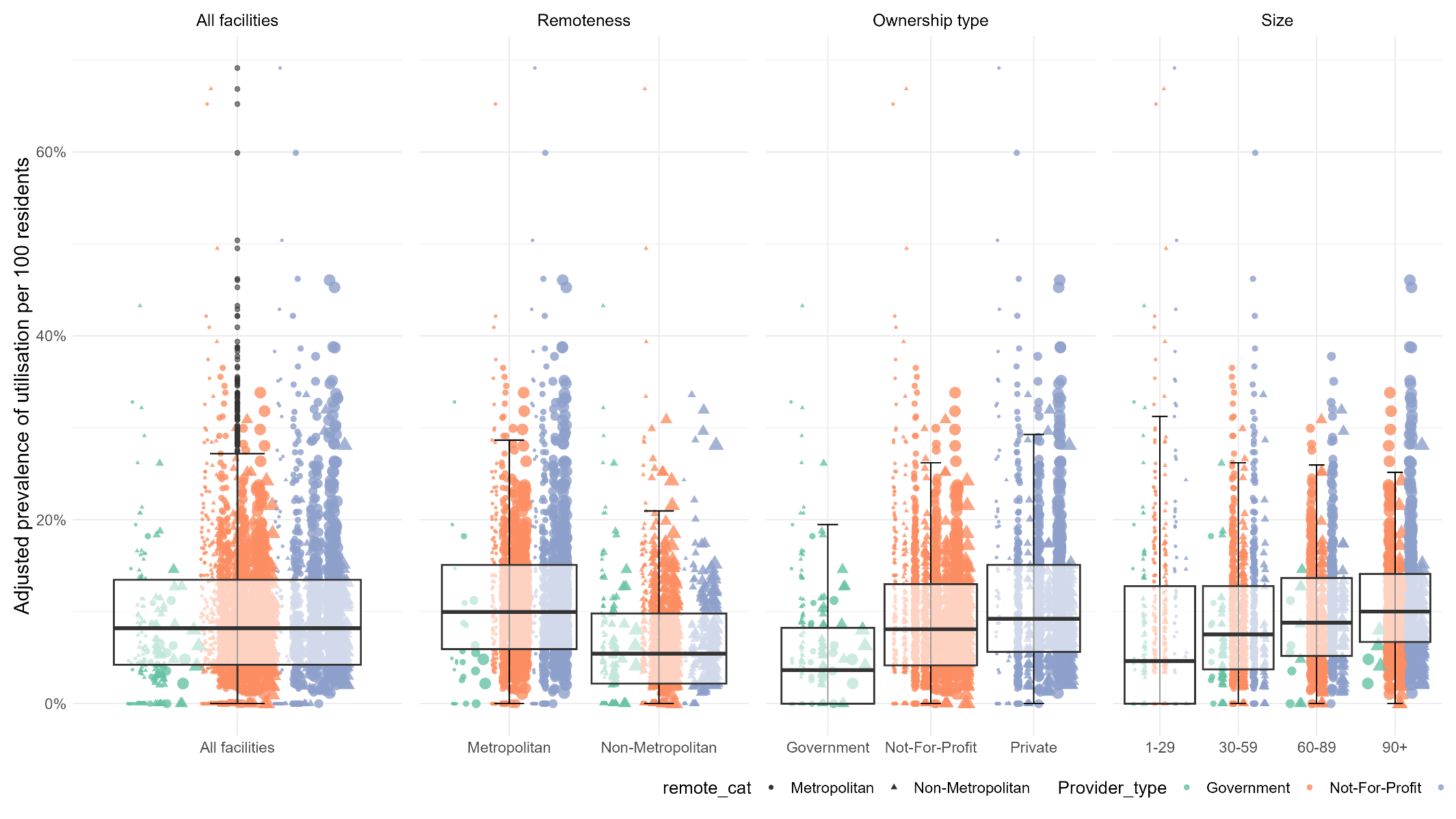

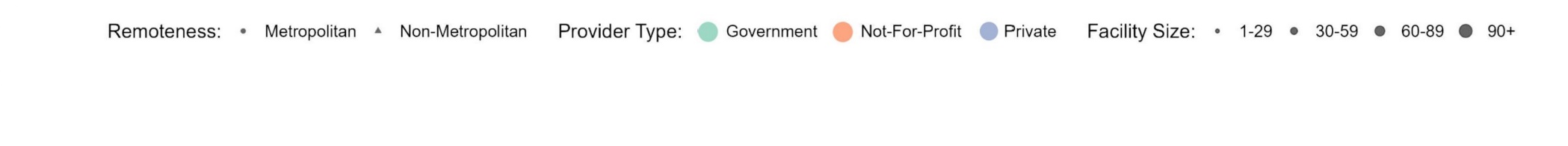


Note. N=2733. On an individual level, utilisation of a service was measures binary with at least one utilisation versus none. The adjusted prevalence of utilisation in 100 residents can thus be understood similar to a percentage of residents who got the service, adjusted by resident-mix in terms of age, sex, and comorbidities. Each data point represents one service home. The shape of a point represents service home remoteness (dots=metropolitan, triangles=non-metropolitan). The size of a data point represents the service home size. The colour represents the ownership type. Data points classified as outliers are represented with grey dots mirroring their utilisation level above or below the whiskers.

**Supplemental Figure 9. Combined strip chart and box-whisker plots visualising the adjusted prevalence of Continuity of Care – Known Category in 100 residents of residential aged care homes in 2019 overall and by home characteristics.**


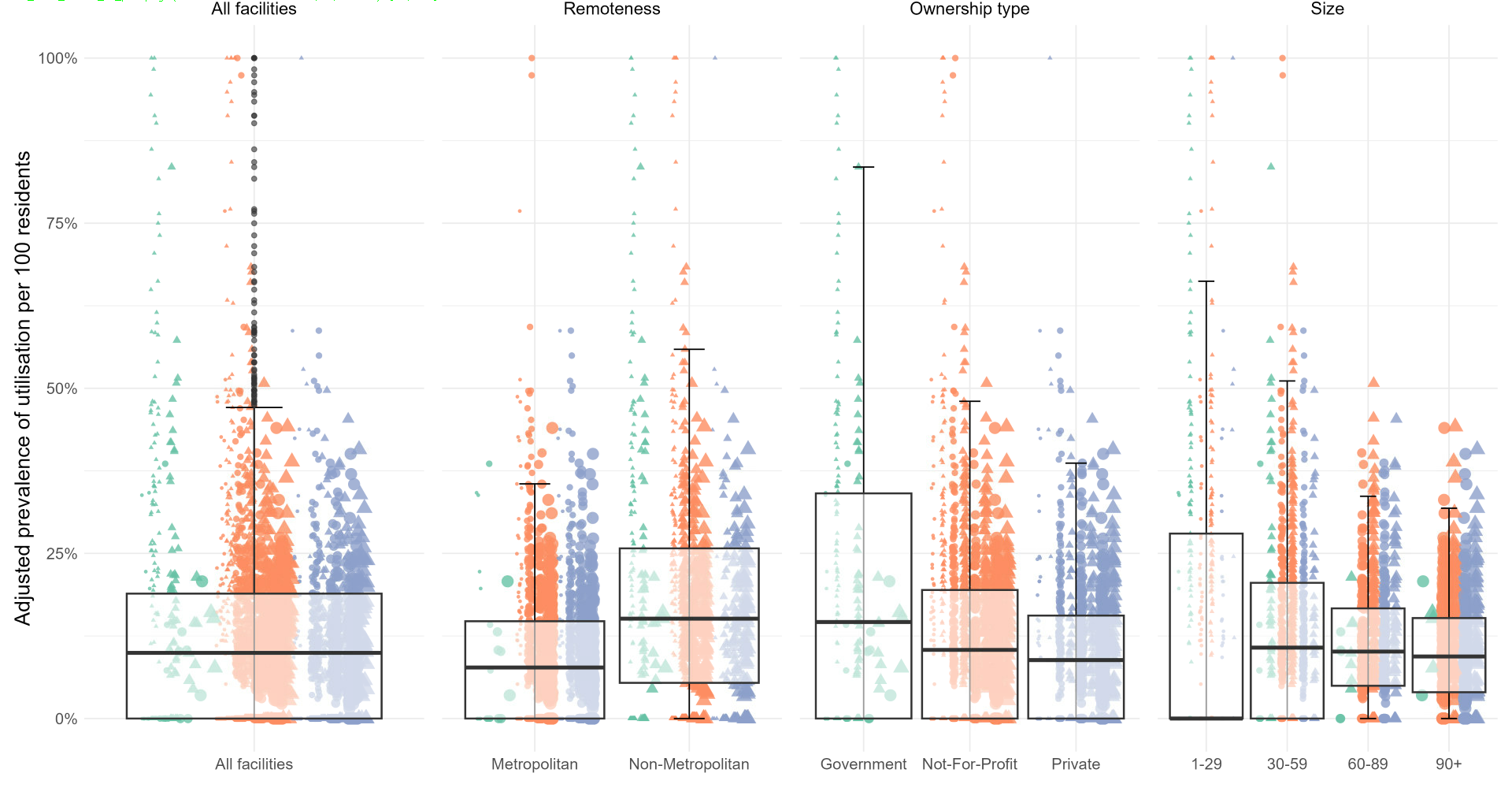

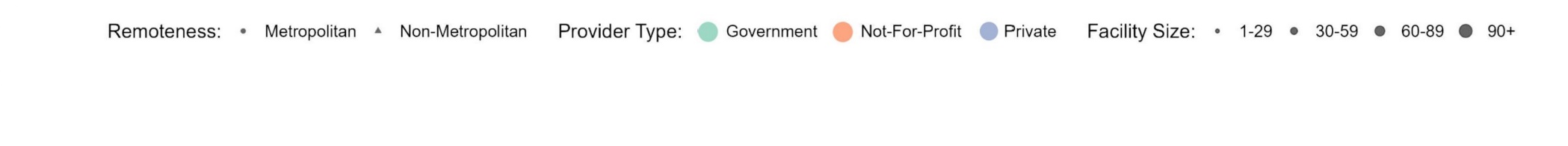


Note. N=2733. On an individual level, utilisation of a service was measures binary with at least one utilisation versus none. The adjusted prevalence of utilisation in 100 residents can thus be understood similar to a percentage of residents who got the service, adjusted by resident-mix in terms of age, sex, and comorbidities. Each data point represents one service home. The shape of a point represents service home remoteness (dots=metropolitan, triangles=non-metropolitan). The size of a data point represents the service home size. The colour represents the ownership type. Data points classified as outliers are represented with grey dots mirroring their utilisation level above or below the whiskers.

**Supplemental Figure 10. Combined strip chart and box-whisker plots visualising the adjusted prevalence of utilisation of General GP/MP attendances in 100 residents of residential aged care homes in 2019 overall and by home characteristics.**


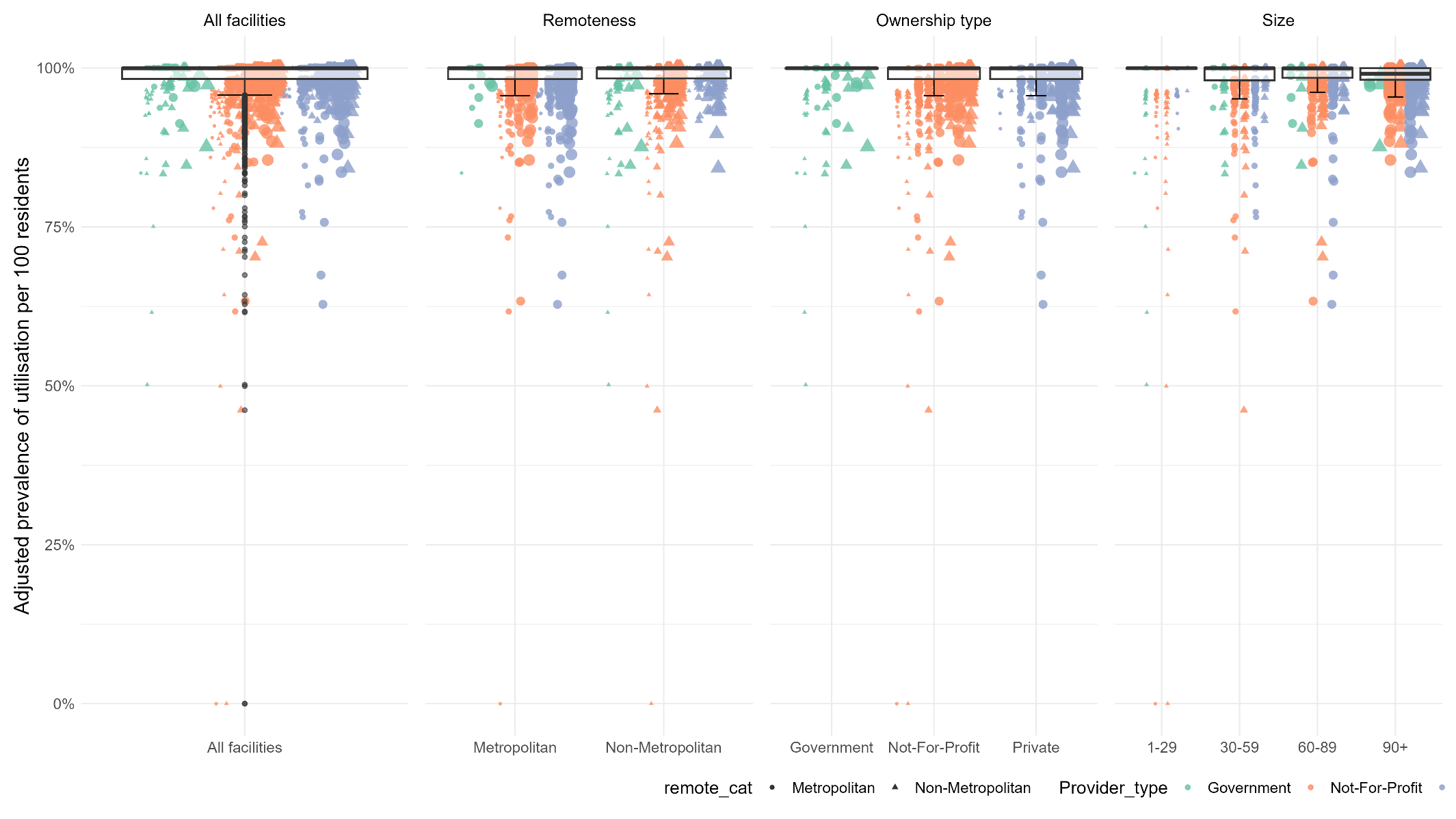

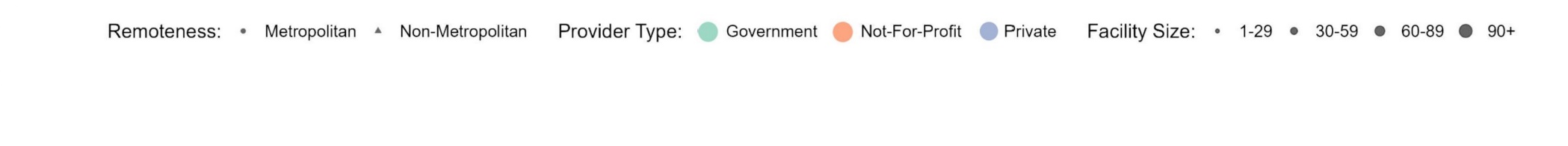


Note. N=2733. On an individual level, utilisation of a service was measures binary with at least one utilisation versus none. The adjusted prevalence of utilisation in 100 residents can thus be understood similar to a percentage of residents who got the service, adjusted by resident-mix in terms of age, sex, and comorbidities. Each data point represents one service home. The shape of a point represents service home remoteness (dots=metropolitan, triangles=non-metropolitan). The size of a data point represents the service home size. The colour represents the ownership type. Data points classified as outliers are represented with grey dots mirroring their utilisation level above or below the whiskers.

**Supplemental Figure 11. Combined strip chart and box-whisker plots visualising the adjusted prevalence of utilisation of General GP/MP attendances associated with the Practice Incentives Program in 100 residents of residential aged care homes in 2019 overall and by home characteristics.**


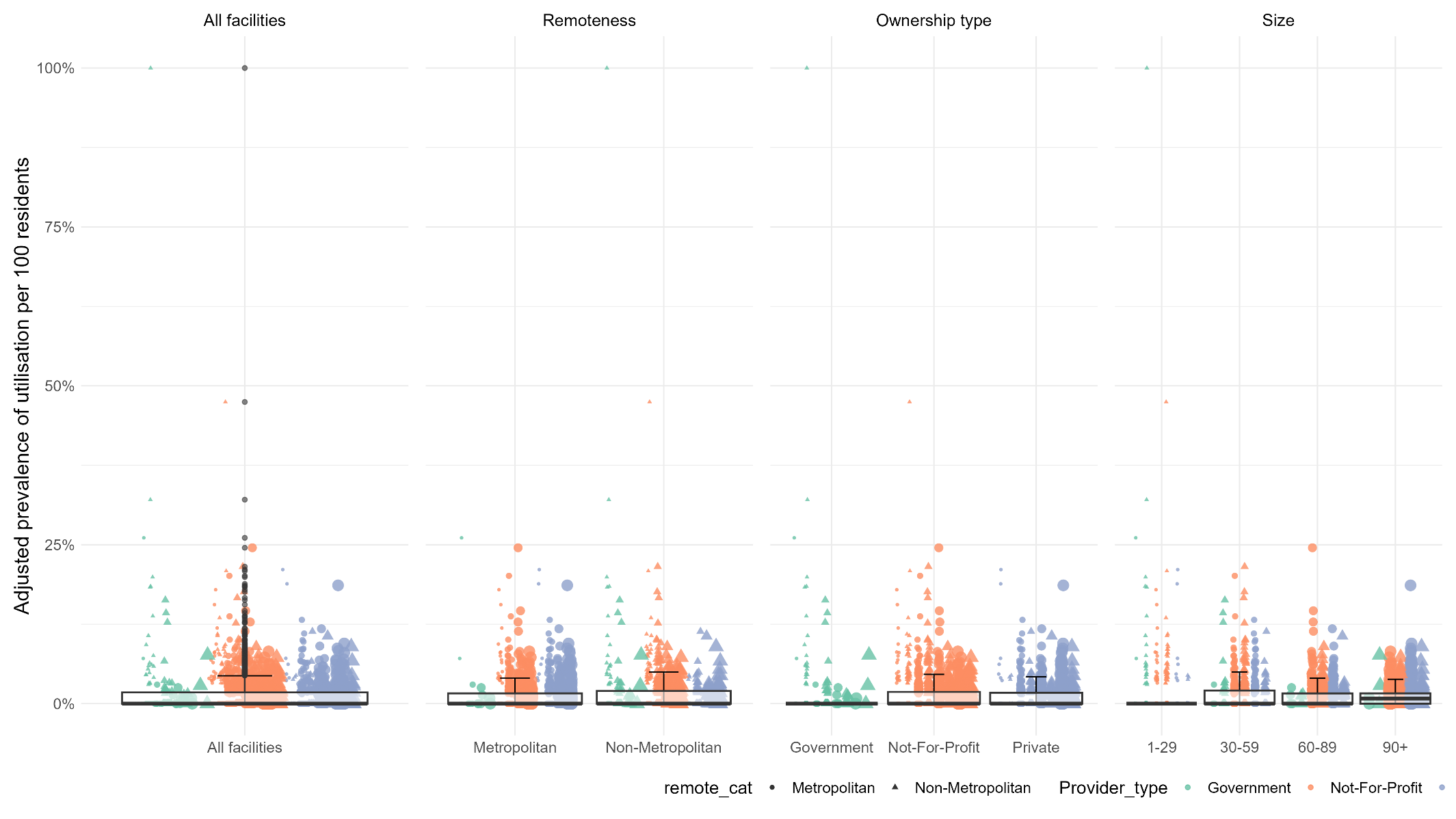

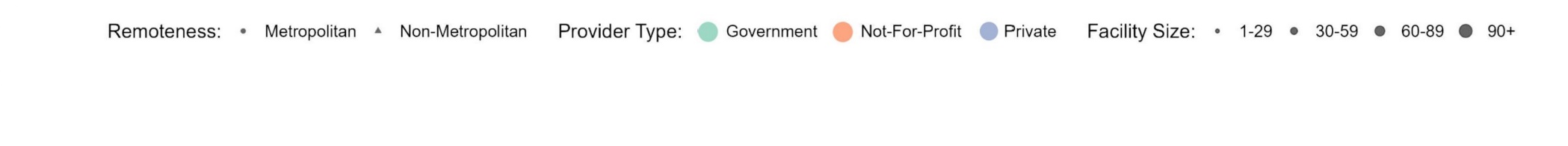


Note. N=2733. On an individual level, utilisation of a service was measures binary with at least one utilisation versus none. The adjusted prevalence of utilisation in 100 residents can thus be understood similar to a percentage of residents who got the service, adjusted by resident-mix in terms of age, sex, and comorbidities. Each data point represents one service home. The shape of a point represents service home remoteness (dots=metropolitan, triangles=non-metropolitan). The size of a data point represents the service home size. The colour represents the ownership type. Data points classified as outliers are represented with grey dots mirroring their utilisation level above or below the whiskers.

**Supplemental Figure 12. Combined strip chart and box-whisker plots visualising the adjusted prevalence of utilisation of Pain Medicine Specialists in 100 residents of residential aged care homes in 2019 overall and by home characteristics.**


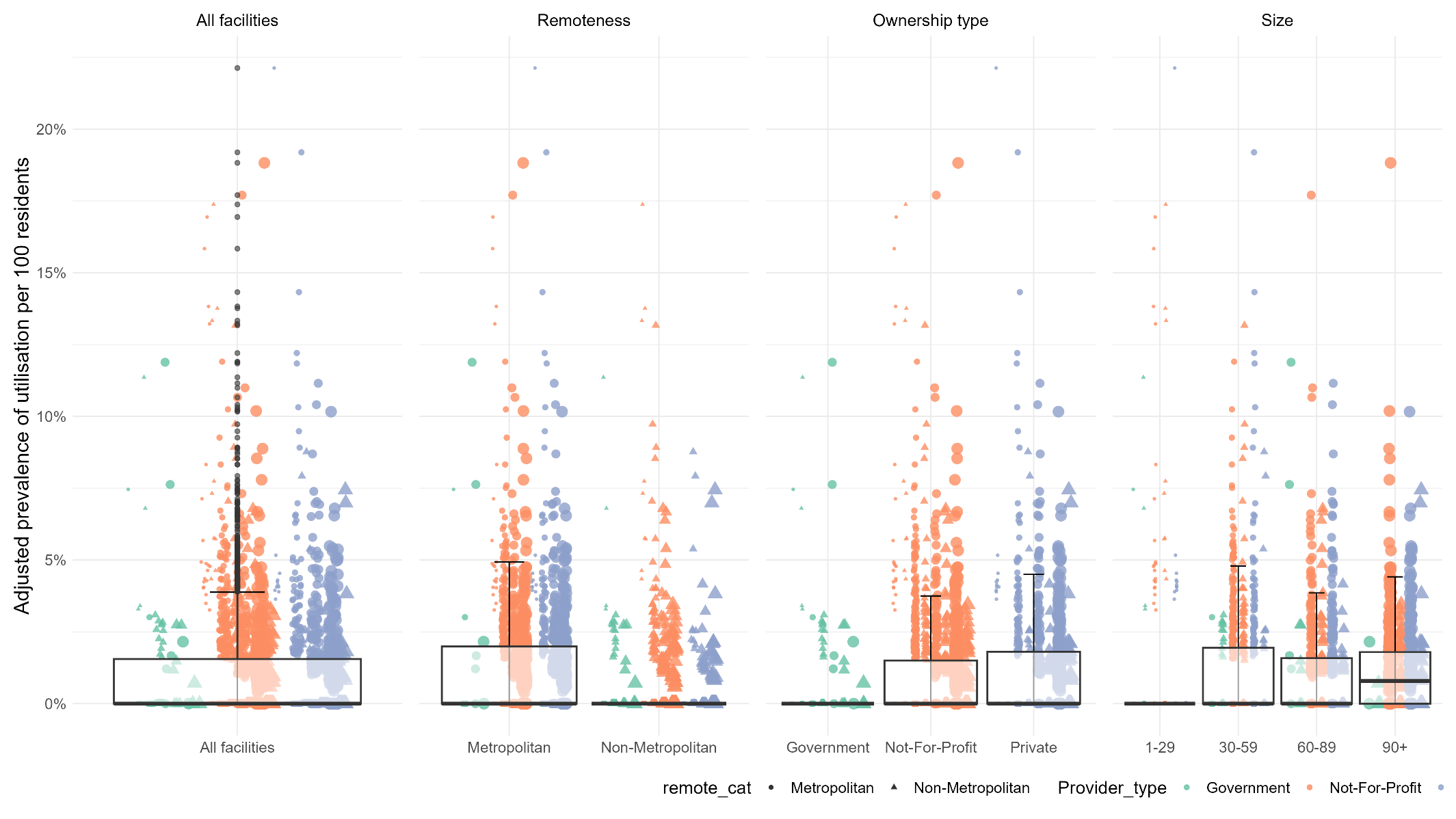

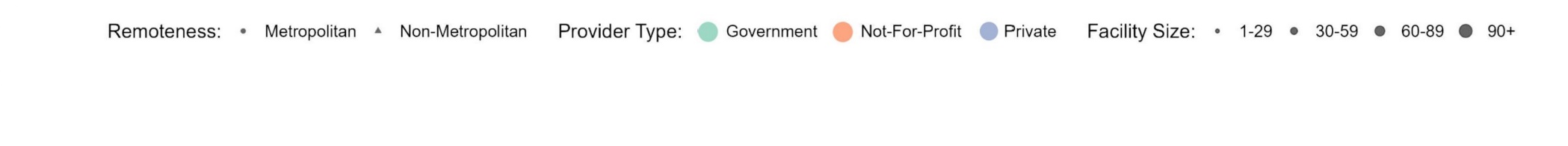


Note. N=2733. On an individual level, utilisation of a service was measures binary with at least one utilisation versus none. The adjusted prevalence of utilisation in 100 residents can thus be understood similar to a percentage of residents who got the service, adjusted by resident-mix in terms of age, sex, and comorbidities. Each data point represents one service home. The shape of a point represents service home remoteness (dots=metropolitan, triangles=non-metropolitan). The size of a data point represents the service home size. The colour represents the ownership type. Data points classified as outliers are represented with grey dots mirroring their utilisation level above or below the whiskers.

**Supplemental Figure 13. Combined strip chart and box-whisker plots visualising the adjusted prevalence of utilisation of Psychiatry attendances in 100 residents of residential aged care homes in 2019 overall and by home characteristics.**


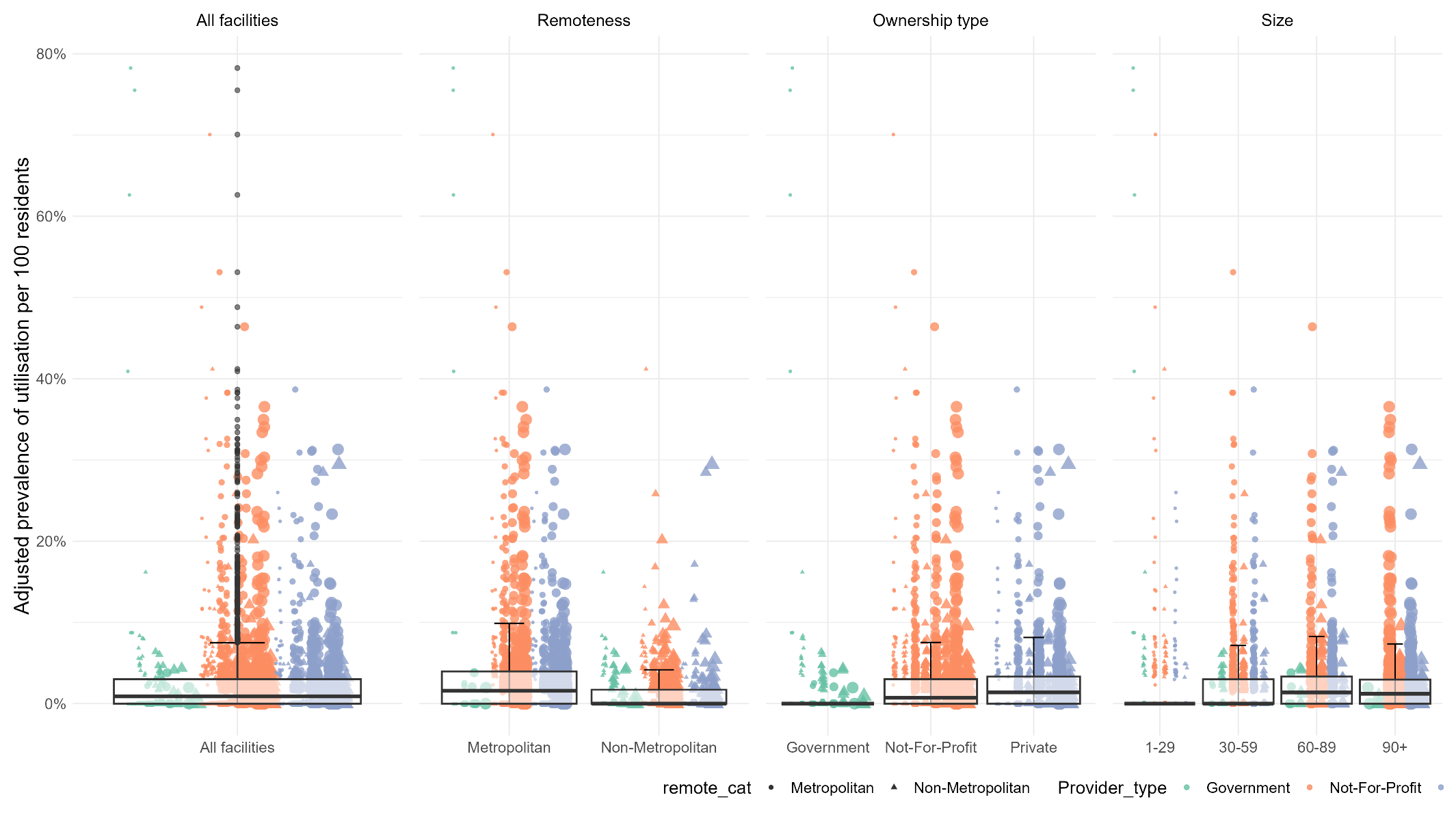

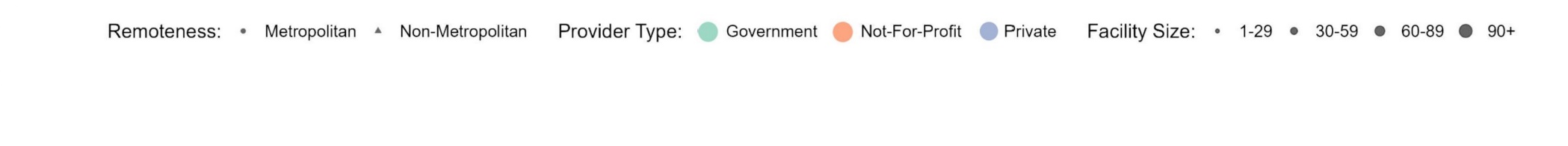


Note. N=2733. On an individual level, utilisation of a service was measures binary with at least one utilisation versus none. The adjusted prevalence of utilisation in 100 residents can thus be understood similar to a percentage of residents who got the service, adjusted by resident-mix in terms of age, sex, and comorbidities. Each data point represents one service home. The shape of a point represents service home remoteness (dots=metropolitan, triangles=non-metropolitan). The size of a data point represents the service home size. The colour represents the ownership type. Data points classified as outliers are represented with grey dots mirroring their utilisation level above or below the whiskers.

**Supplemental Figure 14. Combined strip chart and box-whisker plots visualising the adjusted prevalence of utilisation of Psychological Therapy attendances in 100 residents of residential aged care homes in 2019 overall and by home characteristics.**


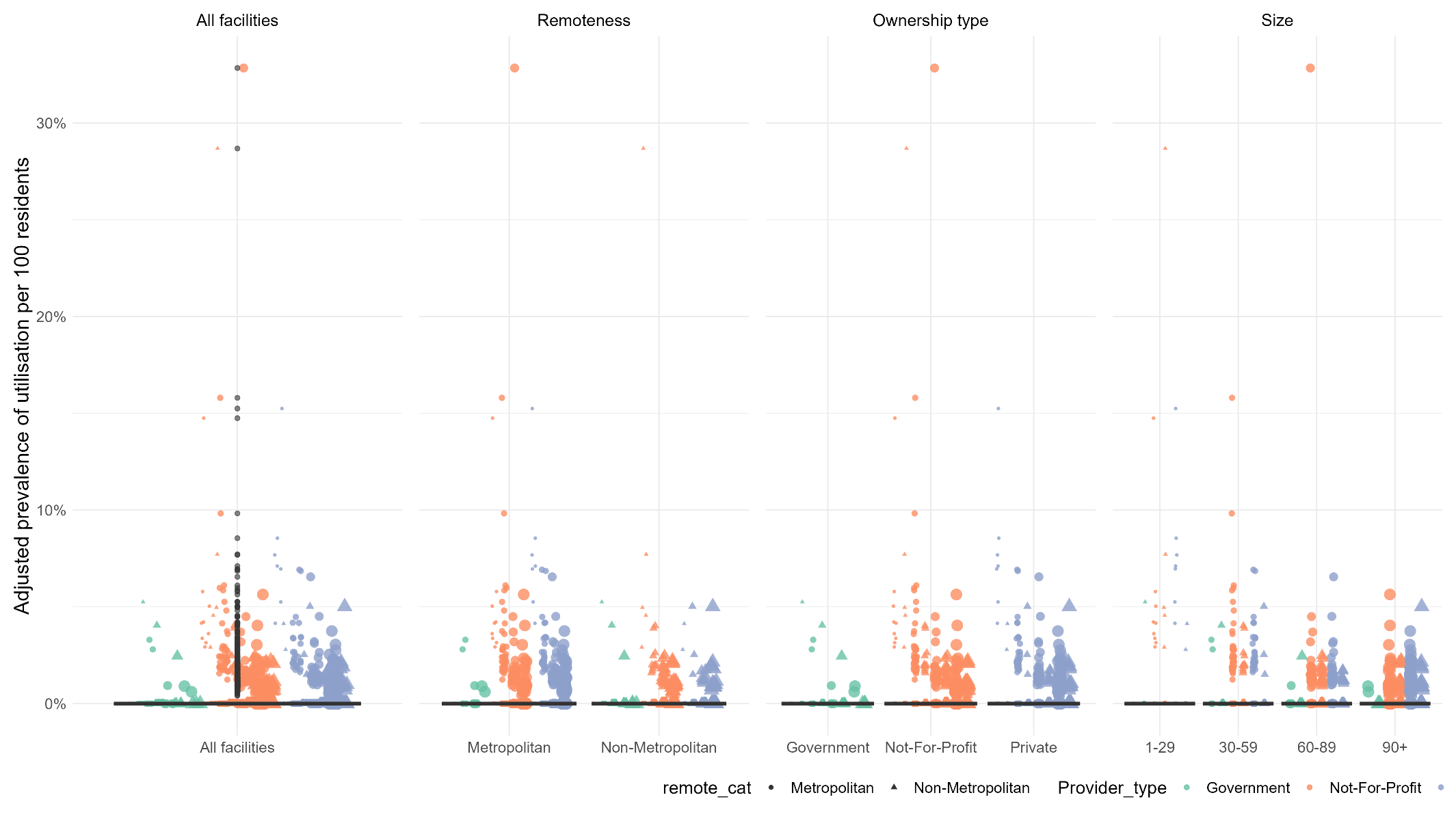

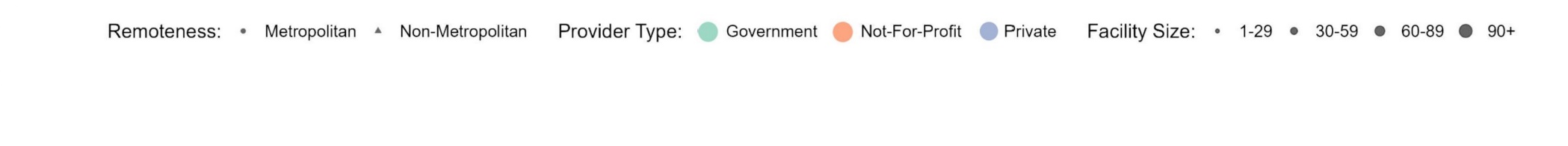


Note. N=2733. On an individual level, utilisation of a service was measures binary with at least one utilisation versus none. The adjusted prevalence of utilisation in 100 residents can thus be understood similar to a percentage of residents who got the service, adjusted by resident-mix in terms of age, sex, and comorbidities. Each data point represents one service home. The shape of a point represents service home remoteness (dots=metropolitan, triangles=non-metropolitan). The size of a data point represents the service home size. The colour represents the ownership type. Data points classified as outliers are represented with grey dots mirroring their utilisation level above or below the whiskers.

**Supplemental Figure 15. Combined strip chart and box-whisker plots visualising the adjusted prevalence of utilisation of Focussed Psychological Strategies attendances in 100 residents of residential aged care homes in 2019 overall and by home characteristics.**


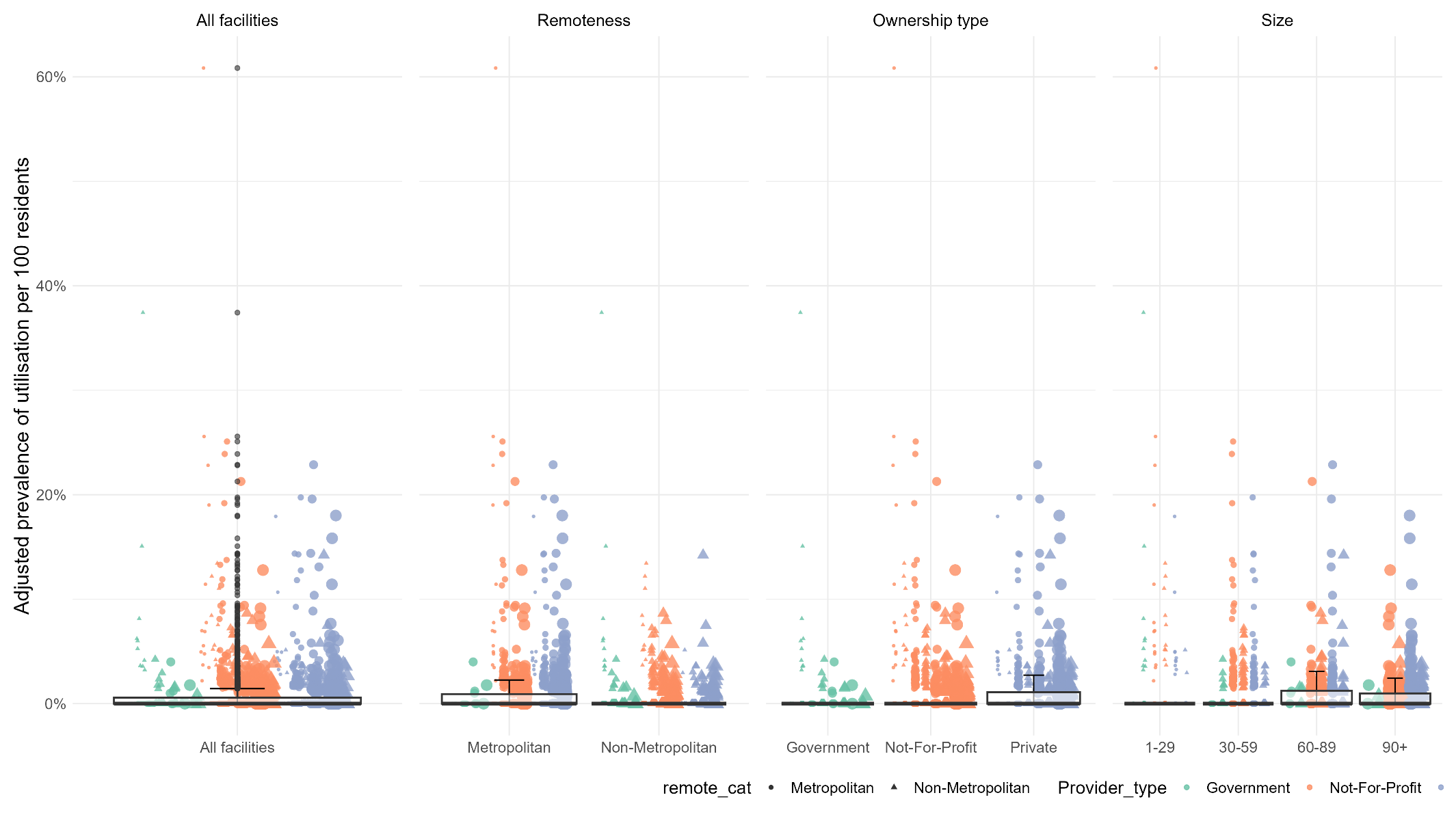

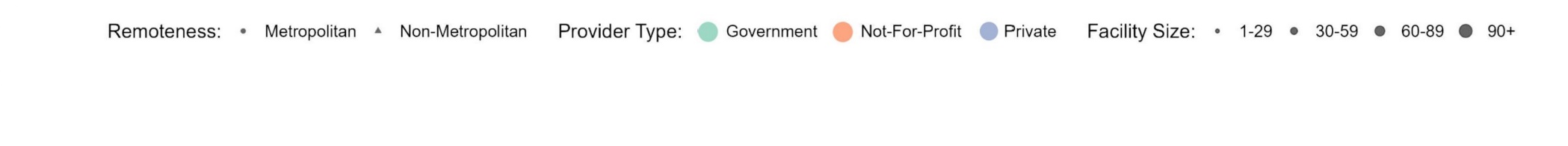


Note. N=2733. On an individual level, utilisation of a service was measures binary with at least one utilisation versus none. The adjusted prevalence of utilisation in 100 residents can thus be understood similar to a percentage of residents who got the service, adjusted by resident-mix in terms of age, sex, and comorbidities. Each data point represents one service home. The shape of a point represents service home remoteness (dots=metropolitan, triangles=non-metropolitan). The size of a data point represents the service home size. The colour represents the ownership type. Data points classified as outliers are represented with grey dots mirroring their utilisation level above or below the whiskers.

**Supplemental Figure 16. Combined strip chart and box-whisker plots visualising the adjusted prevalence of utilisation of GP Mental Health attendances in 100 residents of residential aged care homes in 2019 overall and by home characteristics.**


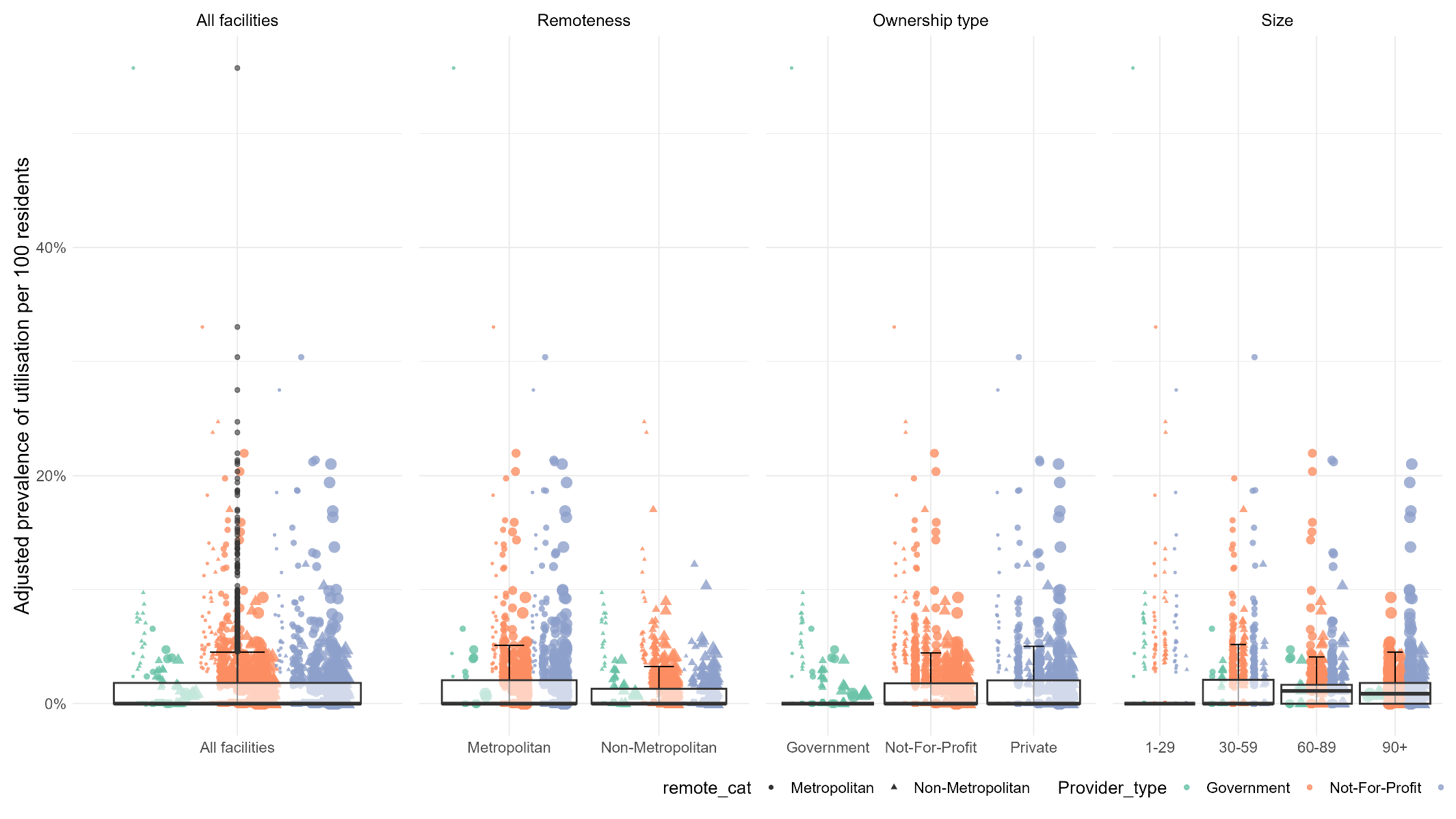

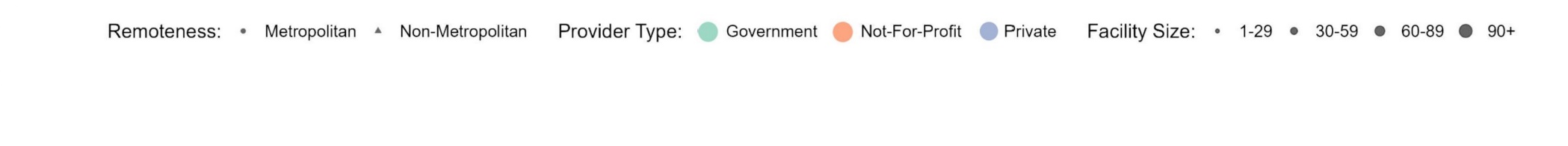


Note. N=2733. On an individual level, utilisation of a service was measures binary with at least one utilisation versus none. The adjusted prevalence of utilisation in 100 residents can thus be understood similar to a percentage of residents who got the service, adjusted by resident-mix in terms of age, sex, and comorbidities. Each data point represents one service home. The shape of a point represents service home remoteness (dots=metropolitan, triangles=non-metropolitan). The size of a data point represents the service home size. The colour represents the ownership type. Data points classified as outliers are represented with grey dots mirroring their utilisation level above or below the whiskers.
